# CRL4-Cereblon complex in Thalidomide Embryopathy: a translational investigation

**DOI:** 10.1038/s41598-020-57512-x

**Published:** 2020-01-21

**Authors:** Thayne Woycinck Kowalski, Julia do Amaral Gomes, Gabriela Barreto Caldas Garcia, Lucas Rosa Fraga, Vanessa Rodrigues Paixao-Cortes, Mariana Recamonde-Mendoza, Maria Teresa Vieira Sanseverino, Lavinia Schuler-Faccini, Fernanda Sales Luiz Vianna

**Affiliations:** 1 Postgraduate Program in Genetics and Molecular Biology, Universidade Federal do Rio Grande do Sul (UFRGS), Porto Alegre, Brazil; 2 Laboratory of Medical and Population Genetics, Universidade Federal do Rio Grande do Sul (UFRGS), Porto Alegre, Brazil; 3 National Institute of Population Medical Genetics (INAGEMP), Porto Alegre, Brazil; 4 Genomic Medicine Laboratory, Centro de Pesquisa Experimental, Hospital de Clínicas de Porto Alegre (HCPA), Porto Alegre, Brazil; 5 National System of Information on Teratogenic Agents (SIAT), Medical Genetics Service, Hospital de Clínicas de Porto Alegre (HCPA), Porto Alegre, Brazil; 6 Complexo de Ensino Superior de Cachoeirinha (CESUCA), Cachoeirinha, Brazil; 7 Department of Morphological Sciences, Institute of Health Sciences, Universidade Federal do Rio Grande do Sul (UFRGS), Porto Alegre, Brazil; 8 Institute of Biology, Universidade Federal da Bahia (UFBA), Salvador, Brazil; 9 Institute of Informatics, Universidade Federal do Rio Grande do Sul (UFRGS), Porto Alegre, Brazil; 10 Bioinformatics Core, Centro de Pesquisa Experimental, Hospital de Clínicas de Porto Alegre (HCPA), Porto Alegre, Brazil; 11 School of Medicine - Pontificia Universidade Catolica do Rio Grande do Sul, Porto Alegre, Brazil; 12 Immunobiology and Immunogenetics Laboratory, Departamento de Genética, Universidade Federal do Rio Grande do Sul (UFRGS), Porto Alegre, Brazil

**Keywords:** Next-generation sequencing, Molecular biology

## Abstract

The Cereblon-CRL4 complex has been studied predominantly with regards to thalidomide treatment of multiple myeloma. Nevertheless, the role of Cereblon-CRL4 in Thalidomide Embryopathy (TE) is still not understood. Not all embryos exposed to thalidomide develop TE, hence here we evaluate the role of the CRL4-Cereblon complex in TE variability and susceptibility. We sequenced *CRBN*, *DDB1*, *CUL4A*, *IKZF1*, and *IKZF3* in individuals with TE. To better interpret the variants, we suggested a score and a heatmap comprising their regulatory effect. Differential gene expression after thalidomide exposure and conservation of the CRL4-Cereblon protein complex were accessed from public repositories. Results suggest a summation effect of *Cereblon* variants on pre-axial longitudinal limb anomalies, and heatmap scores identify the *CUL4A* variant rs138961957 as potentially having an effect on TE susceptibility. *CRL4-Cereblon* gene expression after thalidomide exposure and CLR4-Cereblon protein conservation does not explain the difference in Thalidomide sensitivity between species. In conclusion, we suggest that CRL4-Cereblon variants act through several regulatory mechanisms, which may influence CRL4-Cereblon complex assembly and its ability to bind thalidomide. Human genetic variability must be addressed not only to further understand the susceptibility to TE, but as a crucial element in therapeutics, including in the development of pharmacogenomics strategies.

## Introduction

Thalidomide and its derivatives, lenalidomide and pomalidomide, are potent immunomodulatory (IMiDs), antiangiogenic and anti-inflammatory drugs^1^. Identified in 1961, Thalidomide Embryopathy (TE) is the consequence of embryonic exposure to thalidomide and is mainly characterized as limb reduction defects, although it can affect almost every organ and system^2,3^.

**Figure 1:**
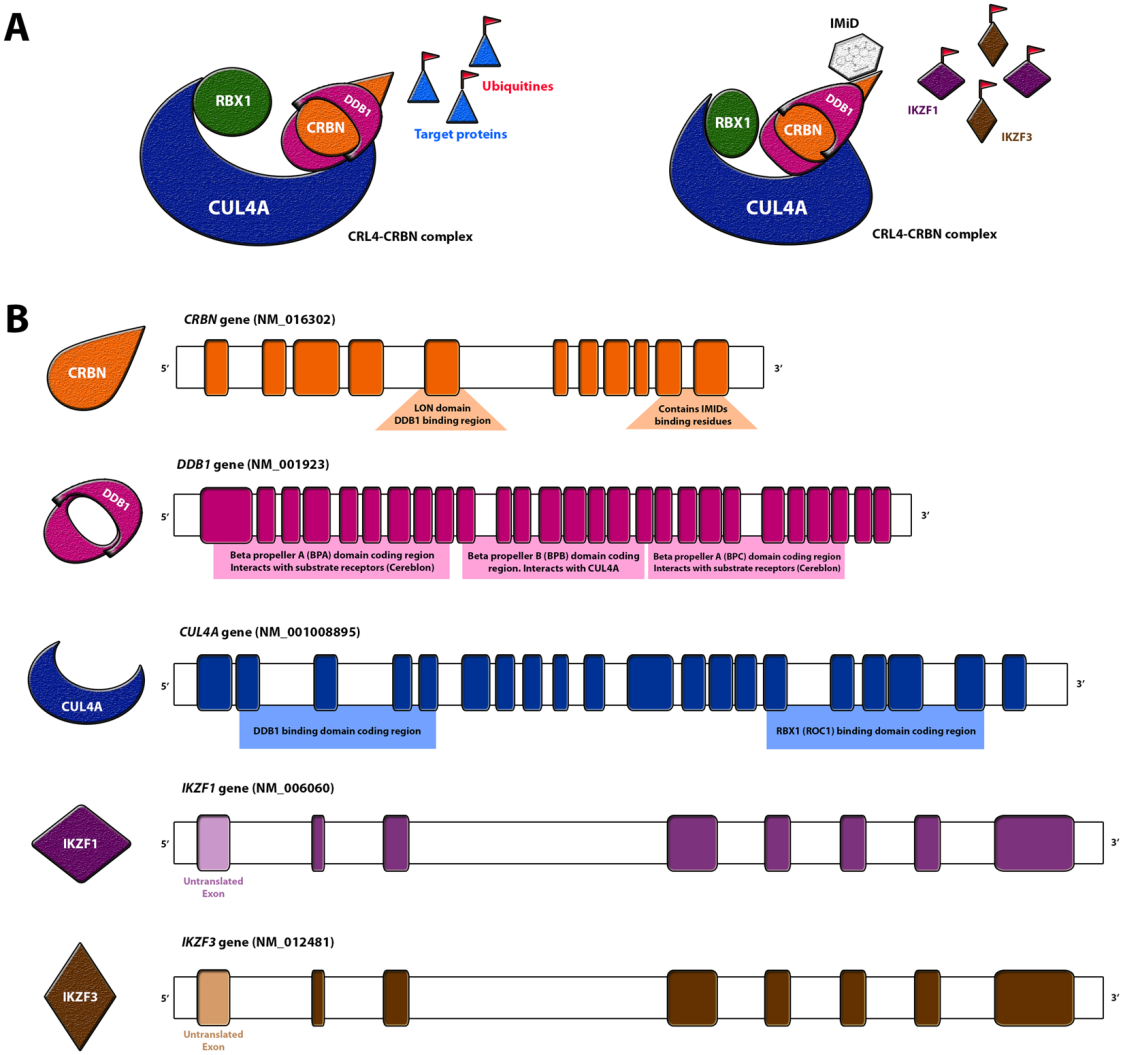
CRL4 complex assembly in the presence and absence of immunomodulatory drugs, and its respective coding genes. (**A**) Graphic representation of the CRL4 complex in the absence (left) and presence (right) of thalidomide, where IKZF1 and IKZF3 are targeted; (**B**) CRL4 complex genes and encoded domains. IMiD = immunomodulatory drugs.

In 2010, Ito *et al*. demonstrated thalidomide’s ability to bind the Cereblon protein, a substrate receptor of an E3-ubiquitin-ligase complex (CRL4^CRBN^)^4^. CRL4 complexes have four subunits: the cullin protein CUL4A acts as a molecular scaffold in the assemble of the subunits; the adaptor protein DDB1 anchors the substrate receptor; the ROC1 protein, with the RING domain, catalyzes substrate ubiquitination; the substrate receptor, a DDB1 and CUL4-associated factor (DCAF), here represented by Cereblon (CRBN), targets the proteins to be ubiquitinated (Fig. 1A)^5,6^.

An early hypothesis suggested that thalidomide-CRL4^CRBN^ binding might interfere in the ubiquitination of the target proteins by inhibiting CRL4^CRBN^ complex formation^4^, affecting downstream limb development genes and resulting in TE^4^. However, it was later identified that the IMiDs cause an allosteric modification of the CRL4^CRBN^ complex, resulting in ubiquitination and degradation of transcription factors (TFs) Ikaros (IKZF1) and Aiolos (IKZF3)(Fig. 1B)^7,8^. IKZF1 and IKZF3 are highly expressed in multiple myeloma, which lead to the discovery of the IMiDs as important therapeutic drugs in this condition^7,9^. Additional proteins potentially affected by the IMiDs binding CRL4^CRBN^ have since been identified (Table 1); any effect on proteins might be IMiD-dependent or simply intensified by the drugs exposure. Nevertheless, the only identified therapeutic effect of the IMiDs remains the reduced expression of IKZF1 and IKZF3 in multiple myeloma^9,10^.

**Table 1:** Literature main findings for CRL4^CRBN^-thalidomide interaction and induced protein effect.

Pubmed ID	Protein Interaction	IMiD tested in the study	Is the mechanism IMiD dependent?	IMiD-protein interaction effect*
27142104	AGO2	lenalidomide	yes	alters expression.
26990986	GS	none	no	
27294876	CD147	none, Thal, Len, Pom	no	competition for CRBN binding reduces expression
27294876	MCT1	none, Thal, Len, Pom	no	competition for CRBN binding reduces expression
26021757	CLC1	none	no	
19295130	DDA1	none	no	
24292623	IKZF1	Thal, Len, Pom	yes	induced degradation
24292623	IKZF3	Thal, Len, Pom	yes	induced degradation
27468689	TAB2	none	no	
27468689	TRAF6	none	no	
25043012	MEIS2	lenalidomide	no	block MEIS2 binding to CRBN
21232561	AMPKA1	none	no	
23026050	PSMB4	none	no	
27601648	RABGEF1	lenalidomide	no	prevents association to CRBN
31591562	TP63	thalidomide	yes	neosubstrate of thalidomide-CRBN
30067223	SALL4	Thal, Len, Pom	yes	thalidomide-CRBN induced degradation (C2H2 motif)

Thal: thalidomide; Len: lenalidomide; Pom: pomalidomide; *differential mechanism in presence or absence of the IMiD.

Experiments by Ito *et al*. suggested that thalidomide-CRL4^CRBN^ binding might be the primary mechanism of thalidomide teratogenicity^4^. Since then, the evaluation of thalidomide-Cereblon binding and CRL4^CRBN^ complex has been the focus of most research efforts into potential therapeutic use, especially with regards to multiple myeloma. Our group has previously studied *CRBN* gene exons that encode the thalidomide-Cereblon binding region in individuals with TE to show that thalidomide-CRBN region is very conserved^10^. However, many gaps still remain in our understanding of the role of CRL4^CRBN^ in TE.

Complex genetic conditions are often very challenging to evaluate because they are not a result of a single mutation, rather the aggregation of many low risk genetic variants, mainly in regulatory regions, combined with environmental influences^11^. In common, complex disorders, strategies including genome wide association studies (GWAS) and polygenic risk scores help in the identification and interpretation of low risk variants^12,13^. Rare, complex conditions cannot be evaluated by the same strategies because the relative low sample sizes impede a robust statistical approach. Low risk, regulatory variants, are mainly bypassed or poorly interpreted.

The aim of this study is to assess the sequence of *CRBN*, *CUL4A*, *DDB1*, *IKZF1*, and *IKZF3* genes in Brazilian individuals with TE, in order to establish a molecular profile for each individual. We devised a unique score and a heatmap results system for robust interpretation of regulatory variants, and conducted methylation analyses of the *CRBN* gene to search for possible epigenetic marks caused by thalidomide. We also conducted a secondary data approach, investigating the differential gene expression (DGE) of the listed targets in embryonic cells and tissues after exposure to thalidomide. We then performed protein conservation analyses across different species that have well characterized TE in order to assess the different sensitivity to thalidomide in these organisms. Finally, through systems biology, we evaluated Gene Ontologies (GO) to understand the role of CRL4^CRBN^ genes during embryonic development.

## Results

### Thalidomide Embryopathy sample represents generations of survivors

Thirty-five individuals with TE, born between 1959 and 2010 were included in this study. Individuals presented upper and/or lower limbs anomalies. Pre-axial longitudinal defects, mainly characterized by malformations in the first digit or intercalary transverse anomalies (phocomelia) were identified; none of the subjects were diagnosed with amelia. Additional external anomalies included eye, ear and other craniofacial malformations. Internal anomalies were mainly cardiovascular and genitourinary. A complete description of individuals clinical characteristics is available in previous research from our group^3^ and in Table S1.

### Gene panel sequencing reveals extremely conserved coding regions

In order to evaluate variants of CRL4 genes, next-generation sequencing was performed using Ion PGM technology (ThermoFisher, USA). Briefly, a gene panel was designed to comprise of all exons, 50 bp of adjacent introns and untranslated regions (5’UTR and 3’UTR). The average depth of sequencing coverage was 491.5X, with at least 100X in 92.44% of the bases, and at least 20X in 97.73% of the regions sequenced.

**Figure 2:**
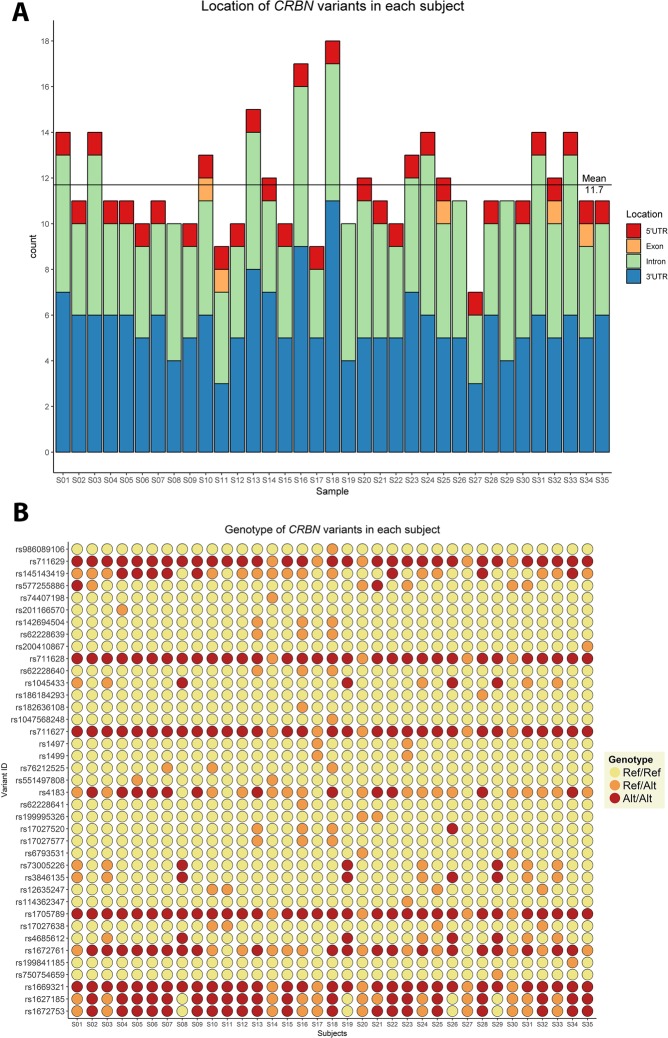
Distribution of *CRBN* variants in subjects with Thalidomide Embryopathy, by gene position and genotypes. (**A**) Number of variants in the *CRBN* gene in each subject with TE, and their respective gene location; (**B**) Genotypes of the *CRBN* variants in each subject with TE. Yellow: Ref/Ref – individual does not present the variant; Orange: Ref/Alt – individual is heterozygous for the variant; Red: Alt/Alt – individual is homozygous for the variant.

**Figure 3:**
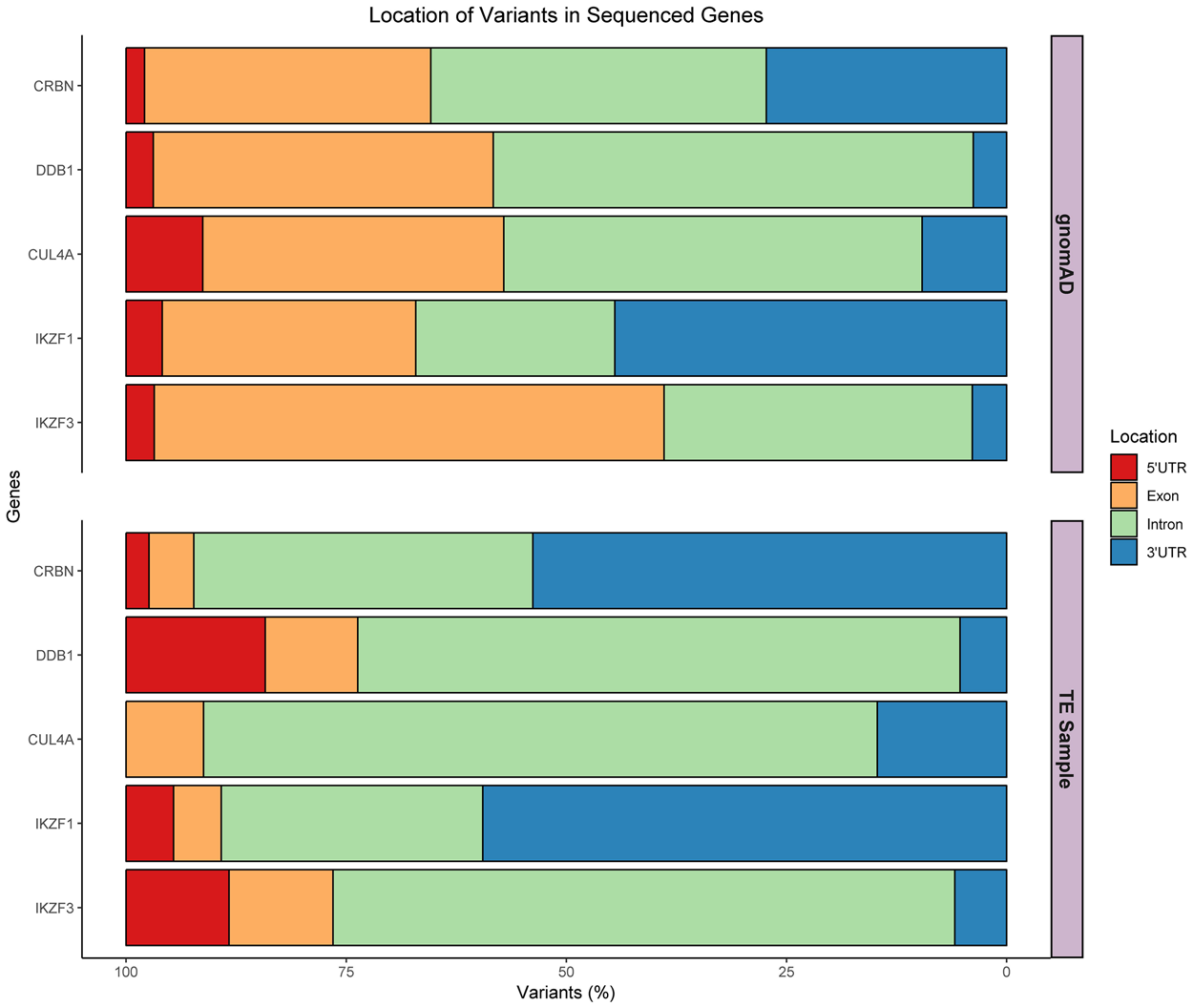
Variants in the Non-Finnish European individuals of the gnomAD database compared to those in the TE sample. Comparison of the variant location distributions between gnomAD and TE, and the percentage of total gene variants at each location.

A total of 145 variants were identified across the five genes (Table S2). *CUL4A* presented three exonic variants, whilst the other four genes, *CRBN*, *DDB1*, *IKZF1*, and *IKZF3* presented only two coding variants; 7.5% of variants are detected in coding regions. *CRBN* and *IKZF1* had the highest number of variants, most located in the 3’UTR region. *CRBN* presented the highest mean of variants per individual (Fig. 2A); the genotype for each subject is represented in Fig. 2B (for *DDB1*, *CUL4A*, *IKZF1*, *and IKZF3* genotypes, see Figs. S1–S4). Three new variants were identified, two in *IKZF1* and one in *DDB1*. The low number of exonic variants in all genes, and the amount of 3’UTR alterations in *CRBN* and *IKZF1* in the sample is remarkable. Moreover, according to the population database, *DDB1* presented the highest number of variants; in our TE sample this was the second most conserved gene. A description of the number of variants identified for each gene and their location can be in Fig. 3.

To verify whether the distribution of identified variants across the genes was similar to a comparison sample, we assessed the variants in the same genomic regions of a gnomAD non-Finnish European sample (subjects without congenital anomalies). This analysis suggested the occurrence of variants between the two samples was statistically different (p < 0.001).

### Extensive in silico functional predictions suggest some variants may affect gene regulatory mechanisms

Missense variants rs2302757 (*CUL4A*, NM_001008895) and rs112301322 (*IKZF3*, NM_012481) were evaluated in SIFT and PolyPhen-2 tools and were predicted to be “tolerated”. Analysis of all nine synonymous variants in SILVA algorithm categorized them as “likely benign”. The effect of all the polymorphisms was evaluated in MutationTaster, PredictSNP-2 and DANN. Analyses also included alteration of splicing and polyA motifs, TFs and microRNA binding sites, CpG islands sizes and mRNA conformation and stability. Characteristics and details of the predictors used are available in Table S3. The profile of each polymorphism regarding population frequency, linkage disequilibrium and previously associated phenotypes were also evaluated.

In a gnomAD non-Finnish European sample, almost 30% (43/145) of the variants encountered were considered rare (Minor Allelic Frequency, MAF < 0.01). Of the 145 variants encountered, 24 are inserted in CpG island regions. Functional prediction demonstrated that ten of these, of which four are found in the *CUL4A* gene, might cause a creation, disruption or size alteration to the CpG islands. microRNA analysis revealed ten variants altered the binding site for miRNAs expressed in human embryonic stem cells (hESC); none of these variants was located in *IKZF3*.

**Figure 4:**
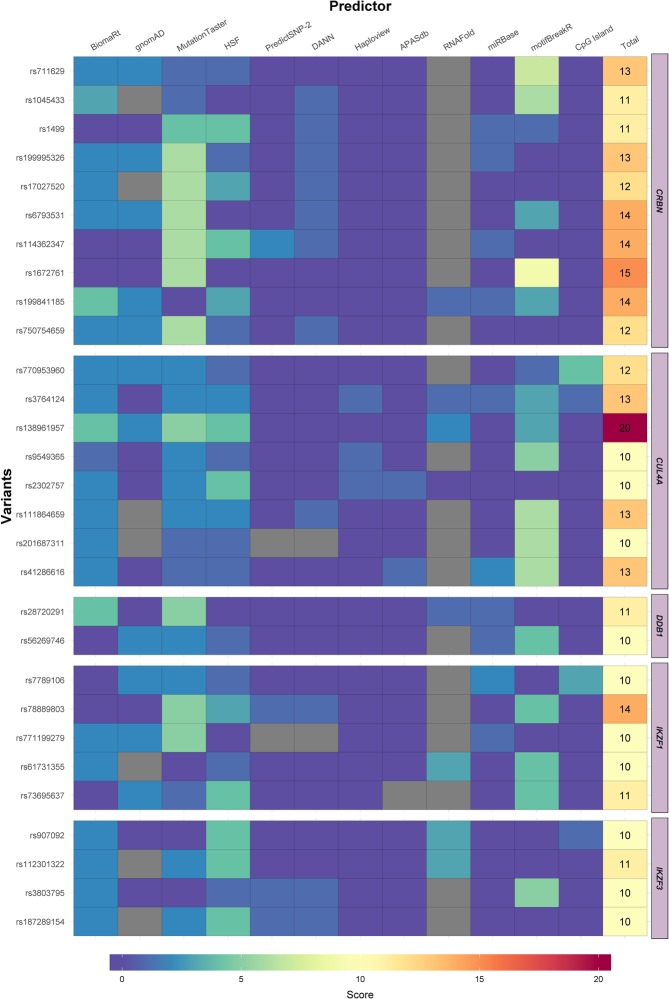
Most relevant variants of *CRBN*, *CUL4A*, *DDB1*, *IKZF1* and *IKZF3* genes according to the heatmap score. Heatmaps for the genes studied, comprised of variants achieving a score of ≥10 in the *in silico* functional prediction.

In order to better comprehend the results of functional predictions and the consequences of the regulatory variants, a score was applied based on the severity of the predicted effect (Table S4). A heatmap then comprised all the functional predictions and all polymorphisms in each gene to give a final score (Figs. S5–S9), ranging from 1 to 20. All variants that presented a score of at least 10 points are comprised in Fig. 4. Each of the three new variants identified, #0000439088 (*DDB1* gene), #0000439108, and #0000439110 (*IKZF1* gene), were scored over 10. The heatmap reveals rs138961957 of *CUL4A* gene as the variant possessing the highest score (20 points), hence the most deleterious to the regulatory mechanisms of all genes examined. rs138961957 is a synonymous c.1392 G > T polymorphism in exon 13 (NM_001008895). This is a very rare variant that was present in two individuals in the TE sample (MAF = 1.499e-05 in European Non-Finnish, according to ExAc database). According to the functional predictions of *motifBreakR* and MutationTaster c.1392 G > T could interfere with splicing and *IKZF1* binding site consensus sequence. It may also affect messenger RNA (mRNA) conformation, resulting in reduced mRNA stability (Fig. S10). Variants rs61731355 of *IKZF1* (Fig. S10), and rs907092 and rs112301322 of *IKZF3*, also synonymous variants, may too alter mRNA conformation. Codon usage in the translation process was evaluated in these three variants, along with*CUL4A* rs138961957. *CUL4A* rs138961957 and *IKZF1* rs61731355 variants lead to incorporation of an alternative codon in comparison to the codon most frequently incorporated, demonstrated both when evaluating gene translation and when accessing the whole human genome (File S2). The other synonymous variation, *IKZF3* rs907092, results in incorporation of the most frequent codon upon evaluation of *IKZF3* gene translation, but incorporation of a less frequent one on evaluation of the whole genome (File S2).

The *CRBN* gene had ten variants with a score of at least ten points. The majority of these polymorphisms scored highly because any splicing site alteration before exon 10 might lead to the loss of the C-terminal region and thalidomide binding site. Moreover, *CRBN* rs711629 also affected *IKZF1* binding site consensus sequence.

IMiDs cause a conformational change in the CRL4^CRBN^ complex, hence it is important to verify the position of variants across the protein domains (Fig. S11). Variants with higher score (black flags) are usually in proximity to exons that do not encode the domains for assembling the CRL4^CRBN^ complex, the exception being the *CUL4A* region that encodes the ROC1 binding site. Additionally, no variant was identified around the IMiDs binding residues.

In summary, a low number of exonic variants were identified in individuals with TE, and most variants are synonymous. Many of the non-coding variants encountered are rare and impact regulatory mechanisms that may influence gene expression and protein function. *CUL4A* variant rs138961957 was considered the most deleterious.

### A high number of variants in individuals with TE are related to pre-axial longitudinal upper limb anomalies

To determine whether the variants identified could have a clinical effect we performed a statistical analysis correlating the variants with the spectrum of congenital anomalies of the individuals with TE. Evaluation was performed (1) considering the stochastic effect of variants in each individual (absolute number of variants); and (2) considering the sum of the scores for each variant.

Results analyzing absolute number of variants were not statistically significant, although the sum of all the variants scores is statistically associated to one group of congenital anomalies in the upper limbs: pre-axial longitudinal defects (p = 0.048), which mainly affects the thumbs. Individuals with these patterns of malformations (n = 10) had a mean score of 228.9 points, whilst subjects with intercalary transverse defects (n = 14), known as phocomelia, had an average score of 195 points (Table S5).The effect is more significant if we look at the score of *CRBN* variants separately: pre-axial longitudinal defects were associated to a score of 94.9 vs. 78 points of the intercalary transverse anomalies (p = 0.021) (Table S5).

To verify whether this effect of stochastic variants is inherent to the studied genes or if it is associated to TE, the rate of incidence of the variants in our TE sample was compared to that of 99 individuals of the 1000Genomes sample (Europeans, CEU group).Individuals with TE present a statistically significant summation effect of variants in the five genes when compared with the control sample. This association is valid both when evaluating the number of variants and when analyzing heatmap scores (Table S6). This effect was also significant when evaluating *CRBN* and *DDB1* separately for total numbers of variants and heatmap scores (Table S6). For *IKZF1*, this association was only statistically significant when evaluating the heatmap scores. The presence *CUL4A* and *IKZF3* variants was not statistically different between the groups (Table S6).

To further explore this association, we divided the TE sample into two groups; individuals with pre-axial longitudinal defects (less severe anomalies, mostly affecting thumbs); individuals with intercalary transverse defects, more severe malformations affecting the long bones and leading to phocomelia. An ANOVA-test was performed between the two TE groups and the 99 individuals of the CEU sample from 1000Genomes. Statistical analysis suggested the statistical association previously identified is probably due to the high score of the individuals with pre-axial upper limb anomalies when compared to the other two groups (Table S7).

Finally, based on heatmap results we evaluated any correlation between the highest ranked variants of our study and genotype and TE endophenotypes. Variant rs1045433, a SNP (NM_016302, c.*1123 A > G) in the 3’UTR with a score of 11 points in our heatmap, was statistically significant, demonstrating a high frequency in individuals with pre-axial limb anomalies. Six individuals with the G allele (two in heterozygosis and four in homozygosis) present pre-axial limb anomalies, only one individual heterozygous for the G SNP presents with an intercalary transverse anomaly (p = 0.004) (Table S8).

### CRL4^CRBN^ complex genes are ubiquitously expressed in developing human limbs

To explain the statistical association between genotypes and pre-axial limb anomalies, we performed a DGE analysis from secondary data obtained from the Gene Expression Omnibus (GEO) database. Samples of the first vs. second-to-fifth digits from the upper limbs of human embryos at embryonic day 44 were obtained from GSE42413^14^. None of the genes evaluated were differentially expressed in the first digit compared to the second-to-fifth digits samples (Table S9).

Despite the lack of association between DGE analyses and the variants identified, we aimed to evaluate whether these genes were highly expressed in the developing human upper limbs. Fig. S12 demonstrates *DDB1* and *RBX1* are highly expressed (>200 transcripts per million, TPM) and that *IKZF3* expression was below the minimal levels of detection (<0.5 TPM). To act as controls, we assessed the expression profiles of genes crucial during limb development such as *SALL4*, *BMP4*, *FGF8*, *FGF10*, and *SHH*; the latter especially highly expression in early stages of limb development. Gene expression levels for *CDH5* (encodes ubiquitously expressed cadherin cell-adhesion protein 5), *PAX6* (paired-box development gene 6, expression restricted to brain, pancreas and eyes), and *TLR4* (toll-like receptor 4, not expressed in this stage of development) were analyzed as negative controls.

From the gene expression profile, it is not possible to rule out a role of the CRL4^CRBN^ complex during upper limb development, indeed it is possible *DDB1* and *RBX1* play important roles in this tissue and stage of development. The role of the CRL4-complex might be independent of *CRBN*, acting with other substrate receptors, for example DCAF proteins.

### CRBN is not methylated in individuals with Thalidomide Embryopathy

It is not known whether thalidomide-Cereblon binding results in epigenetic alterations of the human genome. Since gene panel analysis demonstrated some of the identified gene variants could impact CpG islands size, we evaluated 17 CpG dinucleotides located in the promoter region of *CRBN* by bisulfite sequencing (Fig. S13), including cg20912439 and cg15874300. We did not identify any methylation at these sites in the sample of 35 individuals with TE (Fig. S13). Additionally we assessed a methylation array (GSE48472)^15^ from the GEO database and did not identify methylation at the *CRBN* promoter sites when evaluating human saliva samples.

### Purifying selection explains conservation of the proteins across species

To verify whether the observed gene conservation is present across species differently affected by thalidomide exposure, we performed a comparative analysis of *ROC1* and the five target genes in eight different species: human, rhesus monkey, crab-eating macaque, rabbit, chicken and zebrafish (present typical TE) *versus* mouse and rat (less sensitive to thalidomide, do not present typical TE).

**Figure 5:**
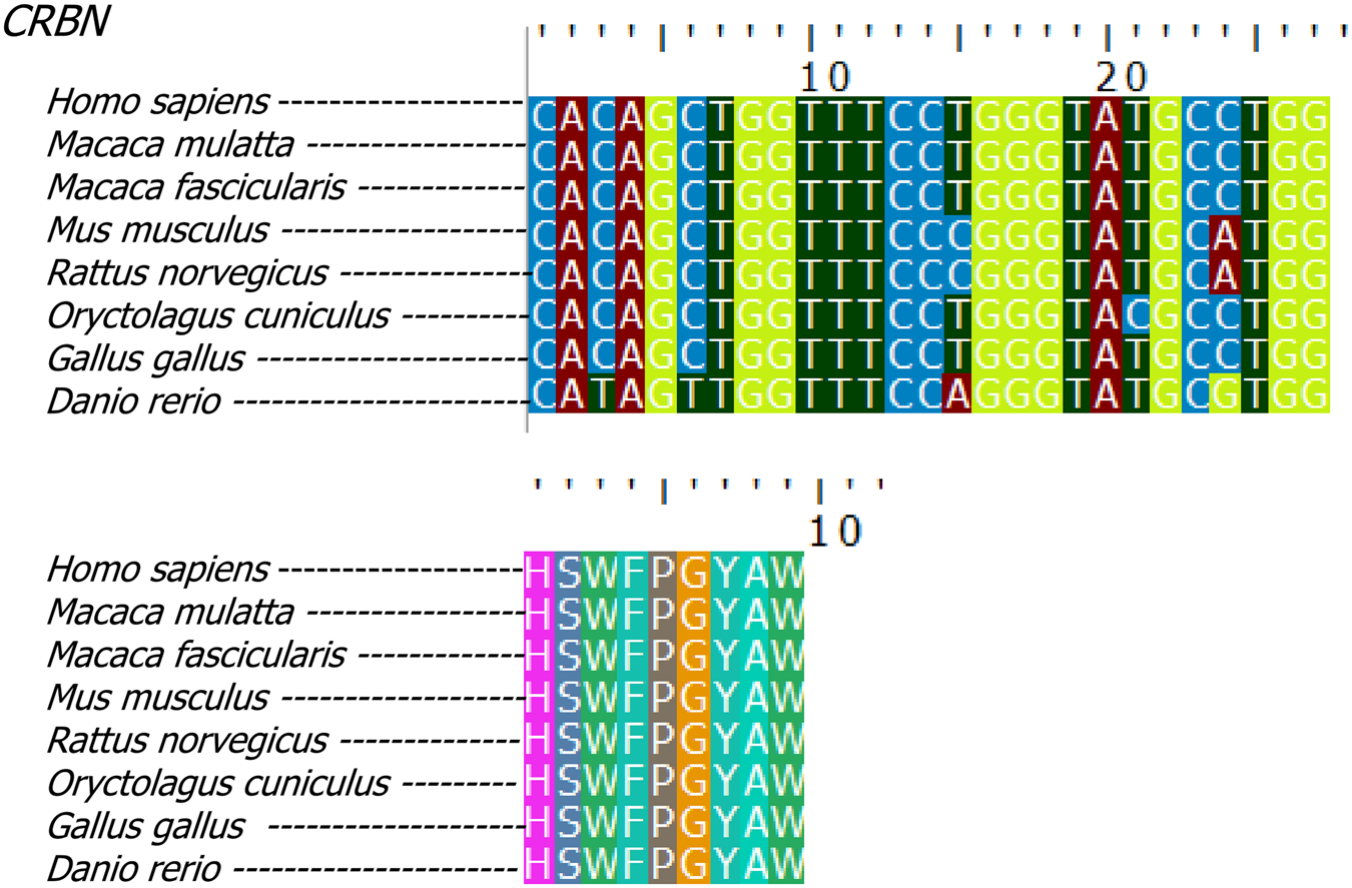
Cereblon gene and protein conservation across different vertebrate species (top) alignment of *CRBN* CULT-domain coding region and translated protein (bottom). Species affected by typical TE are *Homo sapiens* (human), *Macaca mulatta* (Rhesus monkey), *Macaca fascicularis* (crab-eating monkey), *Oryctolagus cuniculus* (rabbit), *Gallus gallus* (chicken) and *Danio rerio* (zebrafish). The rodents *Rattus norvegicus* (rat) and *Mus musculus* (mouse) do not present typical TE, after exposure to thalidomide during embryonic development. Nucleotides (top) and amino acids (bottom) alignment between the eight species studied. Only synonymous mutations are identified in the region. Source: UNIPROT.

On initial NsSites analysis, the P-Values obtained reflect a high state of conservation: ω = 0.0306 at 89% of *CRBN* sites (see Table S10). Across all eight species, *CRBN* complex genes are conserved with the exception of *IKZF3*, which is absent in zebrafish but well conserved among remaining species. Comparison of TE-sensitive species to that of mouse and rat revealed equal conservation between the two groups (Fig. 5, File S1). Purifying selection is the largest force acting on these genes to remove deleterious alleles (P-Value < 1), and according to these results, it is not possible to explain resistance of mouse and rat to TE.

### The effect of Thalidomide on target gene expression is different between mice and humans

In order to determine whether thalidomide affects *CRBN*, *DDB1*, *CUL4A*, *IKZF1*, and *IKZF3* expression in different species, we assessed three studies from the GEO database: mouse (GSE61306)^16^, crab-eating monkey (GSE15542)^17^, and human (GSE63935)^18^. Exposing mouse embryonic stem cells (mESC) to thalidomide or saline solution demonstrates that thalidomide reduces *Cul4a* relative expression for 48 hours post-exposure (p = 0.0068 at 24 hours, p = 0.0195 at 48 hours). *Crbn*, *Ddb1* and *Rbx1* (which encodes Roc1) relative expression is likewise reduced (p = 0.0244, p = 0.0365 and p = 0.0117, respectively). 72 hours after thalidomide exposure none of the genes differs statistically in expression levels compared to control samples (Table S11). In crab-eating monkey secondary data analysis, only *Cul4a* was reduced 6 hours post-thalidomide when compared to the controls(p = 0.0121) (Table S12). Two days and six days after exposure of thalidomide to human pluripotent stem cells (hPSC), the DGE was not statistically different for any of the genes (Table S13).

### Network analysis of the CRL4^CRBN^ demonstrates its role in repair and cancer pathways

**Figure 6:**
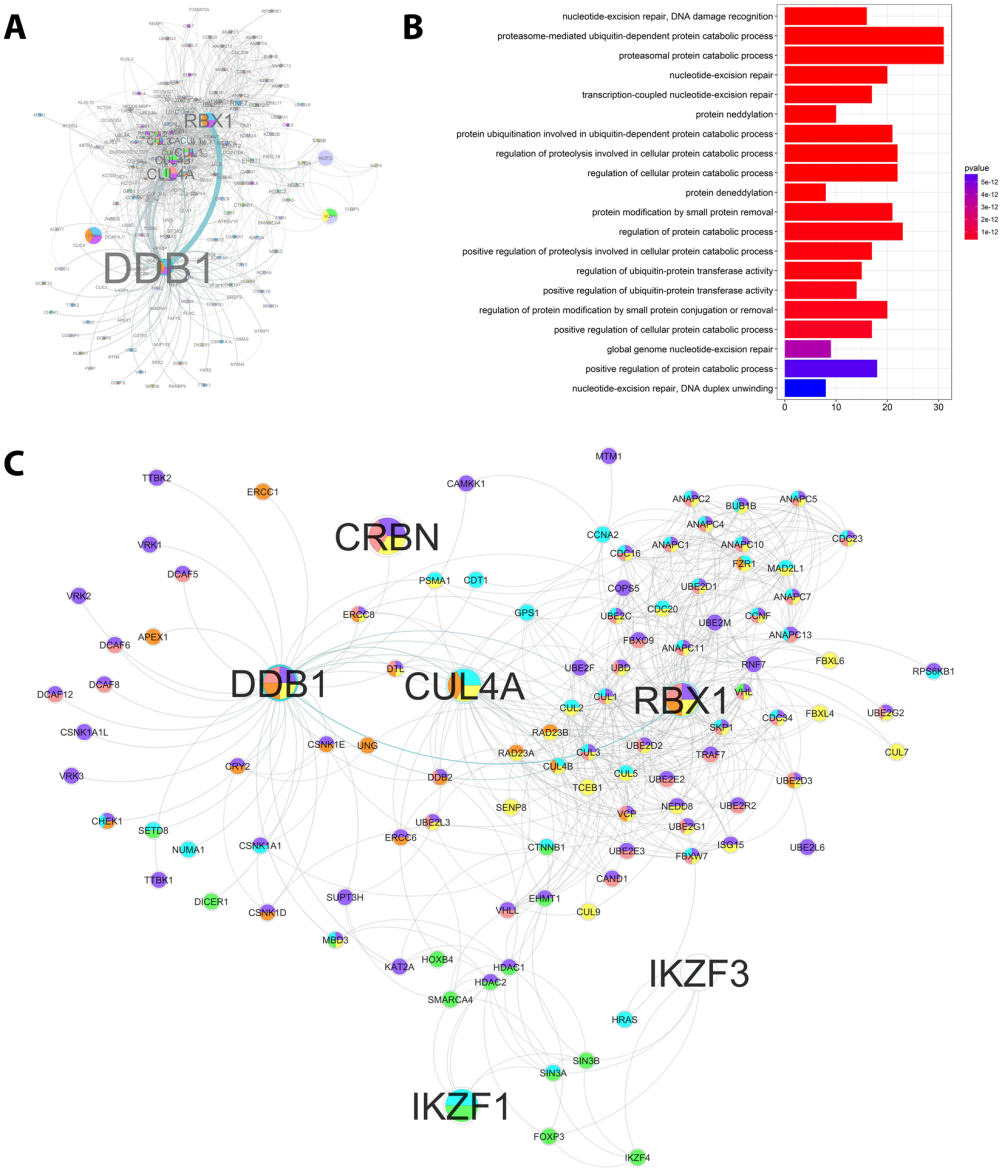
CRL4 complex protein-protein interactions and gene ontologies. (**A**) CRL4 protein-protein interactions (PPI), with letter size corresponding to the number of interactions; (**B**) Gene ontologies (GO) enrichments for the proteins that interact with the CRL4 complex; (**C**) CRL4 complex PPI network representing GO more relevant in terms of TE. Blue: cell cycle; purple: proteolysis; pink: post-translational modification; yellow: ubiquitination; orange: DNA repair; green: gene expression negative regulation.

To help determine the roles of the CRL4^CRBN^ complex during embryonic development, a systems biology network was established. Analysis of protein-protein interactions (PPI) revealed 199 targets of CRBN, CUL4A, DDB1, ROC1, IKZF1, and IKZF3; DDB1 appears to be the main hub of the network (Fig. 6A).

Enrichment analysis of Gene Ontology (GO) and Kyoto Encyclopedia of Genes and Genomes (KEGG) Pathways identified biological processes associated to DNA repair and ubiquitination as the most represented for the CRL4^CRBN^ network (Figs. 6B and S14). Additional associated biological processes identified in analysis of GOs that may aid understanding of the effect of thalidomide on embryonic development are: cell cycle; proteolysis; post-translational protein modification; negative regulation of gene expression (Fig. S14). There was no above-average representation of processes associated with “embryogenesis” or “development” (Fig. 6C).

## Discussion

Genes of the Ubiquitin E3 ligase complex *CUL4A*, *DDB1*, their receptor *CRBN*, and transcription factors *IKZF1*, and *IKZF3*, are well-conserved, especially in the regions that encode main protein domains. It is estimated that around 40% of the children affected by TE died in infancy due to severe internal malformations^19^, and perinatal complications of TE may have caused many more stillbirths. It cannot be determined whether pregnancy loss due to TE complications was associated with deleterious CRL4^CRBN^ complex variants.

Two studies recently demonstrated that Cereblon targets protein SALL4 for degradation in a thalidomide-dependent manner^20,21^, a process which appears to be species-specific when evaluating mice, rabbits and humans, due to missense mutations in the *SALL4* coding sequence, but not in *CRBN*^20,21^. Our results support these studies, demonstrating genetic variation of *CRBN* genetic variation is not sufficient to explain occurrence of TE. *SALL4* mutations in humans cause Duane-radial-ray syndrome^22,23^, a phenocopy of deformities caused by TE. In a previous study from our group, we performed an analysis of *SALL4* in TE individuals and identified 15 variants. Of these 15, ten were located in exons, seven synonymous and three missense^24^. The lack of exonic variants identified in our study prevented the analysis of the effects of combined *SALL4* and *CRBN* variants.

In this study 92.5% of identified variants were located in regulatory regions. The proportion of exonic variants present in individuals with TE was significantly different compared to the gnomAD database sample; TE individuals have much less coding variants than general sample from gnomAD database. This underrepresentation of coding variants might be an effect of the small sample size or might be inherent of the characteristics of these subjects. GWAS reveal, however, almost 90% of disease-associated variants are located outside protein coding regions^11^, and this combined with advancing NGS technologies are promoting studies into regulatory variants and their impact on regulatory elements^25^. The few germinative *CRBN* variants that have been linked to thalidomide responsiveness in treatment of MM are found in regulatory regions^26–28^. Indeed, in a microarray of more than 300,000 exonic SNPs performed in MM patients treated with a chemotherapy scheme comprising thalidomide and bortezomib, no significance was found in the presence of any variant^29^. The continued study of regulatory variants is of great importance towards determining the cause and predictability of thalidomide pharmacogenomic and toxicogenomic effects.

We integrated all generated functional prediction data in a heatmap produced a score for each variant. This methodical approach highlighted those variants more likely to impact TE development, and is useful for further studies of complex disorders with small sample sizes.

In accordance with previous findings^10^, individuals with TE presented a high number of rare variants (MAF < 0.01). Rare variants have been widely studied in complex traits including diabetes^30^, Alzheimer^31^ and cancer^32^, reflecting a shift in human genetic research of multifactorial diseases^33^.Comparison with a European sample of the 1000Genomes project demonstrated individuals with TE present a summation effect of identified variants. The results were similar when evaluating the total number of variants in TE individuals and comparing scores determined in the heatmap. Heatmap scores also suggest synonymous variants may have effects such as altered mRNA conformation and stability and codon usage bias, which influences a series of molecular aspects such as RNA stability, protein folding and gene expression^34^. Indeed, a number of studies have demonstrated the effect of synonymous mutations on efficiency of translation speed and protein folding and a summation effect of synonymous variants may increase the probability of reduced translation efficiency^35,36^. Synonymous mutations have been linked to over 50 genetic diseases^37^, with at least 4% deleterious to splicing motifs^38^. This highlights the importance of further investigation into variants identified in this study, in particular *CUL4A* rs138961957. Statistical analysis revealed no one variant is a determinant for TE incidence, the causative effect linked only to thalidomide. *CRBN* 3’UTR variant rs1045433 is associated with upper limb pre-axial longitudinal anomalies, but not associated with a response to thalidomide treatment^26^. Upper limb pre-axial longitudinal anomalies in upper limbs were associated with higher heatmap scores for multiple genes when evaluating TE individuals only or in comparison with the 1000Genomes European sample. There was no differential expression of analyzed genes in the first digit (pre-axial) compared to second through to fifth, however we cannot rule out differential expression of CRL4^CRBN^ downstream targets. It can be hypothesized that the summation of regulatory variants might influence expression and consequent effect of CRBN, impacting the response of exposure to thalidomide. Studying genome and transcriptome analyses after thalidomide exposure may help confirm or disprove this hypothesis.

We highlight the regulatory effect, leading to alterations in mRNA conformation, of many evaluated variants on the binding site of IKZF1, a TF involved in treatment of MM with thalidomide^7,9^. Although predictions pointed to thalidomide affecting CpG island properties, in the experiments to evaluate epigenetic features of TE individuals we found no methylation in the *CRBN* promoter region.

Reduced expression of evaluated genes in a mESC assay was the only association that suggests an impact of thalidomide on transcription rates. Although mice and rats do not present typical TE, a justification for the use of mESCs is the crucial cross regulation between E3-ligases and deubiquitinating enzymes during pluripotency and differentiation of the stem cells^39^. Additional functions of the CRL4 complex during development include regulation of cell-cycle progression^40^ and genome reprogramming at preimplantation^41^. However, the role of the CRL4^CRBN^ during embryogenesis is not well understood. *CRBN*, *CUL4A*, *DDB1* and *RBX1* are expressed in digits at human embryonic day 44, therefore we cannot rule out a role for CRL4^CRBN^ in normal limb development. It is possible that inhibition of CRL4^CRBN^ complex formation by thalidomide results in severe downregulation of the molecular mechanisms that are CRBN-independent during normal embryogenesis.

CRBN is a well conserved protein across different vertebrate species^4^. Our detection of purifying selection and the high level of gene expression suggests CRBN plays a role in embryogenesis, particularly during brain development. Indeed, *CRBN* is linked to mild mental retardation syndrome^42^; since thalidomide is a synthetic molecule, purifying selection pressures acting on CRBN cannot be explained by its ability to bind thalidomide.

The resulting proteins of the genes included in this study have well characterized roles during ubiquitination and DNA repair processes but no overrepresented GOs during development. A few known targets may be associated with embryogenesis, and a systems biology approach would be a preferred to identify new targets.

This is the first comprehensive study evaluating the CRL4^CRBN^ complex in TE, despite a small sample size. Future studies would ideally include a control group of Brazilians exposed to thalidomide that did not develop TE.

Teratogenesis is a systemic event initiated an exogenous, teratogenic agent such as thalidomide^43^. Independent of affected molecular mechanisms, for example CRBN-binding, angiogenesis and oxidative stress, gene expression is, most likely deregulated upon teratogenic exposure resulting in a cascade of cellular events ultimately impacting signaling pathways^44^. Outcomes of teratogenic exposure might include lethality, occurrence of malformations such as TE, or normal development^45^. One of the predictions for individual response to exposure may be genomic variability. The use of animal models and cell lines are invaluable in determining the molecular mechanisms of a teratogen, but they lack intraspecific genomic variability. The approach in this study is similar to well stablished pharmacogenomics and toxicogenomic methods, although from a teratogenic perspective.

This study aimed to provide insight into the effect of thalidomide on CRL4^CRBN^ complex formation and consequent development of TE. We discovered that coding regions of the genes in this complex are largely comparable to controls in TE affected individuals, and further studies into the effects of the many regulatory variants identified are important in further understanding the development of TE and predicting individual response to thalidomide exposure.

## Materials and Methods

### Ethical issues

These projects are approved by the Committee of Ethics in Research of Hospital of Clinics of Porto Alegre, Brazil (#10-0244, #10-0410, and #17-0248). All methods were carried out in accordance with relevant guidelines and regulations. Informed consent was obtained from all subjects or from a parent and/or legal guardian (subjects under 18).

### Sample collection and DNA extraction

Subjects included in the study were assessed through the Brazilian Association of People with Thalidomide Syndrome (ABPST). Informed consent was obtained from all subjects or from a parent/legal guardian for the subjects under 18 years. Saliva samples were collected with Oragene OG-500 kit (Genotek, USA). DNA extraction was performed according to the manufacturer’s instructions.

### Gene panel analysis

A customized gene panel was designed with Ion Ampliseq (ThermoFisher, USA). All the exons and untranslated regions (5’UTR and 3’UTR) of the genes *CRBN* (NM_016302), *DDB1* (NM_001923), *CUL4A* (NM_001008895), *IKZF1* (NM_006060), and *IKZF3* (NM_012481) were included, as well as 50 bp of adjacent introns. Next generation sequencing was performed with Ion PGM (ThermoFisher, USA) technology with a 316Chip. Non-covered regions were repeated by Sanger sequencing using an ABI 3130XL genetic analyzer. IonReporter software v.5.2 was used for obtaining the variant call file (vcf), evaluating all the alterations encountered in the analyzed sample.

### Variants interpretation and in silico functional prediction

Variants were catalogued through Ion Reporter software v.5.2 (ThermoFisher, USA). Functional prediction was performed using MutationTaster^46^, SIFT^47^, PolyPhen-2^48^, SILVA^49^, RNAFold^50^, miRbase^51^, Human Splicing Finder 3^52^, PredictSNP-2^53^, ApasDB^54^, EnhancerAtlas^55^, MethPrimer^56^, and Haploview v.4.2^57^, according to the developers instructions. Packages *motifbreakR*^58^ and *biomaRt*^59^ were integrated in RStudio v.3.3 for the functional predictions.

### Statistical analysis and figures

Chi-square and t-tests were performed in SPSS v.18 software. Other statistical analyses were performed in R environment (v.1.0.6). Figures were assembled in RStudio v.3 through package *ggplot2*^60^.

### Methylation analysis

Bisulfite conversion in genomic DNA was performed with Cell-to-CpG bisulfite kit (ThermoFisher, USA). Promoter regions were sequenced from genomic DNA. Primers for methylation analyses were designed with MethPrimer^56^. Original and converted DNA was evaluated through Sanger sequencing in an ABI 3130XL genetic analyzer.

### Protein conservation analysis

The sequences were aligned using MUSCLE algorithm in MEGA 7 software. To verify positive selection, the NsSites test, included in PAML 4.9^61^, was applied.

### Differential gene expression analysis

Expression studies were downloaded from Gene Expression Omnibus (GEO) database (NCBI, USA)^62,63^. Secondary data analysis was performed in RStudio v.3.3. Packages *oligo*^64^ and *affy*^65^ were used for microarray analysis, whilst RNAseq data was evaluated through *edgeR*^66^ package. Microarray data was normalized through robust multi-array average (RMA) and RNA seq data by trimmed mean of M-values (TMM). Differential gene expression (DGE) was defined as adjusted P-value < 0.05.

### Systems biology analyses

Protein-protein interaction networks were obtained from STRING database v.10.5 and transferred to Cytoscape software v.3.6. Cytoscape apps BINGO and GOlorize were used for Gene Ontology visual representation. Gene ontology and KEGG pathway enrichment analyses were performed in *clusterProfileR*^67^ package, RStudio v.3.3.

## Supplementary information

Supplementary Info.

## Data Availability

The datasets generated and analyzed during the current study are not publicly available to maintain patient confidentiality. Moreover, this type of request has not been previously approved by participants nor the Human Research Committee. This data could, however, be available (anonymously) from the corresponding author on reasonable request. Gene panel sequencing files have been deposited in the repository Sequence Read Archive (SRA) of the National Center for Biotechnology Information (NCBI), under accession number SRP160424. The new variants identified in the present study were submitted to the Leiden Open Variation Database (LOVD3), under numbers #0000439088 (*DDB1*), #0000439108 and #0000439110 (*IKZF1*). Gene expression analyses were performed with data from public repository Gene Expression Omnibus (GEO) and are available under the following numbers: GSE42413, GSE61306, GSE15542, GSE63935, and GSE48472.
