# Supplementary material for: CRL4-Cereblon complex in Thalidomide Embryopathy: a translational investigation
Source: Sci Rep. 2020 Jan 21;10:851. doi: 10.1038/s41598-020-57512-x (PMC6972723; doi:10.1038/s41598-020-57512-x)
Supplement: Supplementary file 1 — Supplementary Info. [file 41598_2020_57512_MOESM1_ESM.pdf]

Supplementary Tables 1-13

CRL4-CEREBLON COMPLEX IN THALIDOMIDE EMBRYOPATHY: A TRANSLATIONAL INVESTIGATION

Thayne Woycinck Kowalski

Julia do Amaral Gomes

Gabriela Barreto Caldas Garcia

Lucas Rosa Fraga

Vanessa Rodrigues Paixao-Cortes

Mariana Recamonde-Mendoza

Maria Teresa Vieira Sanseverino

Lavinia Schuler-Faccini

Fernanda Sales Luiz Vianna

Table S1: Anomalies and respective frequencies described, for TE individuals

Location/anomaly	Number of cases	Frequency (%)
**Upper limbs (bilateral)**	**24/25**	**96%**
Intercalary transverse	14/25	56%
Preaxial longitudinal	10/25	40%
**Lower Limbs (bilateral)**	**12/25**	**48%**
Intercalary transverse	10/25	40%
Preaxial longitudinal	2/25	8%
**Other External Anomalies**		
Ears	4/26	15,4%
Eyes	7/24	29,2%
Oral Cavity	2/26	7,7%
**Internal Anomalies**		
Neurological Anomalies	4/26	15,4%
Heart	3/26	11,5%
Urinary Tract	2/26	7,7%
Genital Tract	3/26	11,5%
Digestive Tract	1/26	3,8%
Skeletal	4/26	15,4%

Table S2: Variants Identified in Gene Panel

Gene	Chromosome	Transcript (NM)	hg19 Position	ID	Type	Ref/Alt Alleles	Location	MAF (gnomAD NFE)
CRBN	3	NM_016302	3:3190384	rs986089106	SNV	G/C	3'UTR	0.0000667468 (C)
CRBN	3	NM_016302	3:3190451	rs711629	SNV	G/A	3'UTR	0.001 (G)
CRBN	3	NM_016302	3:3190488	rs145143419	INDEL	T/TATTA	3'UTR	0.426 (T)
CRBN	3	NM_016302	3:3190499	rs577255886	INDEL	T/TA	3'UTR	0.082 (TA)
CRBN	3	NM_016302	3:3190582	rs74407198	SNV	C/T	3'UTR	4.00855e-04 (T)
CRBN	3	NM_016302	3:3190636	rs201166570	INDEL	C/CTATT	3'UTR	8.76885e-05 (TATT)
CRBN	3	NM_016302	3:3191257	rs142694504	INDEL	C/CTGGGATCCACT	3'UTR	0.01 (CTGGGATCCACT)
CRBN	3	NM_016302	3:3191290	rs62228639	SNV	C/T	3'UTR	0.01 (T)
CRBN	3	NM_016302	3:3191336	rs200410867	INDEL	T/TTAAAG	3'UTR	0.015 (TAAAG)
CRBN	3	NM_016302	3:3191388	rs711628	SNV	T/A	3'UTR	0.001 (T)
CRBN	3	NM_016302	3:3191404	rs62228640	SNV	A/G	3'UTR	0.01 (G)
CRBN	3	NM_016302	3:3191426	rs1045433	SNV	T/C	3'UTR	0.152 (C)
CRBN	3	NM_016302	3:3191558	rs186184293	SNV	C/T	3'UTR	0.001 (T)
CRBN	3	NM_016302	3:3191665	rs182636108	SNV	A/G	3'UTR	0.002 (G)
CRBN	3	NM_016302	3:3191770	rs1047568248	SNV	G/C	3'UTR	6.67557e-05 (C)
CRBN	3	NM_016302	3:3191776	rs711627	SNV	T/C	3'UTR	0.001 (T)
CRBN	3	NM_016302	3:3191813	rs1497	SNV	T/G	3'UTR	0.026 (G)
CRBN	3	NM_016302	3:3191833	rs1499	SNV	A/G	3'UTR	0.027 (G)
CRBN	3	NM_016302	3:3191956	rs76212525	SNV	G/C	3'UTR	0.058 (C)
CRBN	3	NM_016302	3:3192246	rs551497808	SNV	A/G	3'UTR	0.002 (G)
CRBN	3	NM_016302	3:3192524	rs4183	INDEL	A/ATAAC	3'UTR	0.335 (A)
CRBN	3	NM_016302	3:3194088	rs62228641	SNV	C/T	Intron	0.002 (T)
CRBN	3	NM_016302	3:3194308	rs199995326	SNV	C/T	Intron	6.67379e-05 (T)
CRBN	3	NM_016302	3:3194369	rs17027520	SNV	T/G	Intron	0.010 (G)
CRBN	3	NM_016302	3:3195325	rs17027577	SNV	T/C	Intron	0.01 (C)

CRBN	3	NM_016302	3:3195351	rs6793531	SNV	G/A	Intron	2.66667e-04 (A)
CRBN	3	NM_016302	3:3195369	rs73005226	SNV	A/G	Intron	0.142 (G)
CRBN	3	NM_016302	3:3195574	rs3846135	SNV	C/T	Intron	0.142 (T)
CRBN	3	NM_016302	3:3195850	rs12635247	SNV	C/G/T	Intron	0.025 (G)
CRBN	3	NM_016302	3:3197800	rs11462347	SNV	A/G	Intron	0.025 (G)
CRBN	3	NM_016302	3:3197871	rs1705789	SNV	T/C	Intron	0.001 (T)
CRBN	3	NM_016302	3:3197918	rs17027638	SNV	A/G	Exon	0.020 (G)
CRBN	3	NM_016302	3:3209242	rs4685612	SNV	G/T	Intron	0.141 (T)
CRBN	3	NM_016302	3:3209655	rs1672761	SNV	A/T	Intron	0.197 (A)
CRBN	3	NM_016302	3:3214576	rs199841185	SNV	T/G	Exon	5.37548e-05 (G)
CRBN	3	NM_016302	3:3214635	rs750754659	SNV	G/A	Intron	0.000 (A)
CRBN	3	NM_016302	3:3215954	rs1669321	SNV	A/G	Intron	0.001 (A)
CRBN	3	NM_016302	3:3221060	rs1627185	SNV	G/A/C	Intron	0.210 (G)
CRBN	3	NM_016302	3:3221430	rs1672753	SNV	C/T	Upstream	0.194 (C)
DDB1	11	NM_001923	11:61067254	rs1132797	SNV	T/C	3'UTR	4.02685e-04 (C)
DDB1	11	NM_001923	11:61071331	rs28720346	SNV	C/T	Splicing, Intron	0.001 (T)
DDB1	11	NM_001923	11:61071518	rs201237741	SNV	C/A	Intron	3.42645e-04 (A)
DDB1	11	NM_001932	11:61076372	rs11230664	SNV	T/C	Intron	0.009 (C)
DDB1	11	NM_001923	11:61077474	rs28720330	SNV	T/C	Intron	1.16678e-04 (C)
DDB1	11	NM_001923	11:61077489	rs28720329	SNV	A/G	Intron	1.99840e-04 (G)
DDB1	11	NM_001923	11:61079175	rs11230667	SNV	G/A	Intron	0.004 (A)
DDB1	11	NM_001923	11:61079415	rs28720314	SNV	A/T	Intron	1.16373e-04 (T)
DDB1	11	NM_001923	11:61083715	rs28720301	SNV	A/T	Intron	0.001 (T)
DDB1	11	NM_001923	11:61084180	rs12289370	SNV	A/G	Intron	0.010 (G)
DDB1	11	NM_001923	11:61089037	rs12290116	SNV	T/C	Intron	0.004 (C)
DDB1	11	NM_001923	11:61089771	rs28720291	SNV	C/T	Exon	0.009 (T)
DDB1	11	NM_001923	11:61092991	ND	SNV	G/T	Intron	ND
DDB1	11	NM_001923	11:61093245	rs7108142	SNV	A/G	Intron	0.004 (G)

DDB1	11	NM_001923	11:61097362	rs28720259	SNV	T/C	Intron	0.002 (C)
DDB1	11	NM_001923	11:61099072	rs2230356	SNV	G/A	Exon	0.109 (A)
DDB1	11	NM_001923	11:61100336	rs56269746	SNV	C/G	Upstream	0.001 (G)
DDB1	11	NM_001923	11:61100471	rs369927446	SNV	G/C	Upstream	0.000 (T)
CUL4A	13	NM_001008895	13:113864488	rs970214412	SNV	G/A	Intron	0.000 (A)
CUL4A	13	NM_001008895	13:113872978	rs116151161	SNV	G/T	Intron	0.000 (T)
CUL4A	13	NM_001008895	13:113883738	rs180741517	SNV	G/T	Intron	0.003 (T)
CUL4A	13	NM_001008895	13:113883851	rs115418707	SNV	G/A	Intron	6.65956e-05 (A)
CUL4A	13	NM_001008895	13:113887281	rs2287245	SNV	C/T	Intron	0.001 (T)
CUL4A	13	NM_001008895	13:113888049	rs931459769	SNV	C/T	Intron	0.000 (T)
CUL4A	13	NM_001008895	13:113888201	rs2146408	SNV	T/C	Intron	0.001 (T)
CUL4A	13	NM_001008895	13:113889355	rs2287249	SNV	A/G	Intron	0.234 (G)
CUL4A	13	NM_001008895	13:113889474	rs2287250	SNV	C/T	Intron	0.253 (C)
CUL4A	13	NM_001008895	13:113889482	rs541918654	SNV	G/A	Intron	3.65965e-05 (A)
CUL4A	13	NM_001008895	13:113889499	rs2287251	SNV	A/G	Intron	0.252 (G)
CUL4A	13	NM_001008895	13:113890981	rs772518711	INDEL	AGTC/A	Intron	0.001 (A)
CUL4A	13	NM_001008895	13:113891042	rs2287252	SNV	T/C	Intron	0.001 (C)
CUL4A	13	NM_001008895	13:113891075	rs2287253	SNV	G/A	Intron	0.252 (G)
CUL4A	13	NM_001008895	13:113893728	rs770953960	SNV	C/T	Intron	2.68827e-05 (T)
CUL4A	13	NM_001008895	13:113893729	rs3794422	SNV	G/A	Intron	0.233 (A)
CUL4A	13	NM_001008895	13:113893887	rs3794423	SNV	C/T	Intron	4.67211e-04 (T)
CUL4A	13	NM_001008895	13:113897320	rs3764124	SNV	C/T	Exon	0.251 (T)
CUL4A	13	NM_001008895	13:113899321	rs138961957	SNV	G/T	Exon	6.66311e-05 (T)
CUL4A	13	NM_001008895	13:113899447	rs61967871	SNV	C/A	Intron	0.027 (A)
CUL4A	13	NM_001008895	13:113900197	rs2302756	SNV	G/A	Intron	0.252 (G)
CUL4A	13	NM_001008895	13:113907391	rs9549365	SNV	A/G	Splicing Site, Intron	0.252 (G)
CUL4A	13	NM_001008895	13:113908995	rs374602605	SNV	C/T	Intron	1.99947e-04 (T)

CUL4A	13	NM_001008895	13:113909339	rs2302757	SNV	A/G	Exon	0.2353 (G)
CUL4A	13	NM_001008895	13:113914596	rs9549720	SNV	A/G	Intron	0.001 (A)
CUL4A	13	NM_001008895	13:113914625	rs111864659	SNV	G/A	Intron	0.000 (A)
CUL4A	13	NM_001008895	13:113914812	rs111568611	SNV	C/T	Intron	0.012 (T)
CUL4A	13	NM_001008895	13:113917604	rs150151004	SNV	T/A	Intron	0.004 (A)
CUL4A	13	NM_001008895	13:113917767	rs4907602	SNV	T/C	Intron	0.001 (T)
CUL4A	13	NM_001008895	13:113917979	rs201687311	INDEL	CTGAT/C	3'UTR	0.000 (C)
CUL4A	13	NM_001008895	13:113918184	rs138211925	SNV	T/A	3'UTR	1.99814e-04 (A)
CUL4A	13	NM_001008895	13:113918770	rs11552924	SNV	A/C	3'UTR	0.043 (C)
CUL4A	13	NM_001008895	13:113918897	rs41286616	SNV	G/A	3'UTR	0.004 (A)
CUL4A	13	NM_001008895	13:113919455	rs116011085	SNV	G/A	Downstream	0.000 (A)
IKZF1	7	NM_006060	7:50344389	rs543438548	SNV	C/T	5'UTR	0.000 (T)
IKZF1	7	NM_006060	7:50344474	rs6975767	SNV	A/C	5'UTR	0.378 (C)
IKZF1	7	NM_006060	7:50344544	rs11506039	SNV	A/G	Intron	0.012 (G)
IKZF1	7	NM_006060	7:50443928	rs74828745	SNV	A/G	Intron	0.033 (G)
IKZF1	7	NM_006060	7:50444025	rs113125091	SNV	C/T	Intron	0.080 (T)
IKZF1	7	NM_006060	7:50444040	rs74412507	SNV	G/A	Intron	0.079 (A)
IKZF1	7	NM_006060	7:50450420	rs7789106	SNV	T/C	Intron	0.011 (C)
IKZF1	7	NM_006060	7:50450446	rs113962761	SNV	C/T	Intron	0.085 (T)
IKZF1	7	NM_006060	7:50454736	rs6962370	SNV	A/G	Intron	0.196 (G)
IKZF1	7	NM_006060	7:50454805	rs75866857	SNV	A/G	Intron	0.079 (G)
IKZF1	7	NM_006060	7:50454980	rs56278999	SNV	A/T	Intron	0.078 (T)
IKZF1	7	NM_006060	7:50459405	rs78889803	SNV	G/A	Intron	3.33156e-04 (A)
IKZF1	7	NM_006060	7:50459406	rs771199279	INDEL	CCT/C	Intron	8.96877e-06 (C)
IKZF1	7	NM_006060	7:50467767	rs61731355	SNV	C/A	Exon	0.197 (A)
IKZF1	7	NM_006060	7:50467941	rs61731356	SNV	C/T	Exon	0.047 (T)
IKZF1	7	NM_006060	7:50468334	rs72645704	SNV	C/T	3'UTR	0.051 (T)
IKZF1	7	NM_006060	7:50468685	0000439108*	SNV	A/G	3'UTR	ND

IKZF1	7	NM_006060	7:50468952	rs10278451	SNV	G/T	3'UTR	0.275 (T)
IKZF1	7	NM_006060	7:50469397	rs73114989	SNV	G/C	3'UTR	0.030 (C)
IKZF1	7	NM_006060	7:50469551	rs11552047	SNV	C/T	3'UTR	0.275 (T)
IKZF1	7	NM_006060	7:50469613	rs34234461	INDEL	TA/T	3'UTR	0.048 (T)
IKZF1	7	NM_006060	7:50469710	rs149985644	SNV	C/T	3'UTR	0.002 (T)
IKZF1	7	NM_006060	7:50469751	rs114837302	SNV	C/T	3'UTR	1.99973e-04 (T)
IKZF1	7	NM_006060	7:50469779	rs75168275	SNV	C/A	3'UTR	0.000 (A)
IKZF1	7	NM_006060	7:50469981	rs11980379	SNV	T/C	3'UTR	0.275 (T)
IKZF1	7	NM_006060	7:50470138	rs62447207	SNV	G/T	3'UTR	0.197 (T)
IKZF1	7	NM_006060	7:50470156	rs62447208	SNV	G/C	3'UTR	0.198 (C)
IKZF1	7	NM_006060	7:50470256	rs58184950	SNV	G/A	3'UTR	0.048 (A)
IKZF1	7	NM_006060	7:50470378	rs33999320	INDEL	T/TC	3'UTR	0.275 (C)
IKZF1	7	NM_006060	7:50470604	rs4132601	SNV	T/G	3'UTR	0.275 (G)
IKZF1	7	NM_006060	7:50470813	0000439110*	SNV	G/A	3'UTR	ND
IKZF1	7	NM_006060	7:50471038	rs114737126	SNV	G/A	3'UTR	0.001 (A)
IKZF1	7	NM_006060	7:50471397	rs72645705	SNV	A/G	3'UTR	0.048 (G)
IKZF1	7	NM_006060	7:50471613	rs11980407	SNV	G/A	3'UTR	0.275 (A)
IKZF1	7	NM_006060	7:50472324	rs62445866	SNV	G/A	3'UTR	0.275 (A)
IKZF1	7	NM_006060	7:50472738	rs73695637	SNV	G/A	3'UTR	0.013 (A)
IKZF1	7	NM_006060	7:50472842	rs58923657	SNV	C/T	Downstream	0.275 (T)
IKZF3	17	NM_012481	17:37921899	rs2952141	SNV	C/T	3'UTR	0.000 (C)
IKZF3	17	NM_012481	17:37922804	rs112876941	SNV	A/T	Intron	0.043 (T)
IKZF3	17	NM_012481	17:37922259	rs907092	SNV	G/A	Exon	0.446 (A)
IKZF3	17	NM_012481	17:37933823	rs12709364	SNV	A/G	Intron	0.043 (A)
IKZF3	17	NM_012481	17:37944411	rs34291217	SNV	C/A	Intron	0.043 (A)
IKZF3	17	NM_012481	17:37944482	rs35088469	SNV	C/T	Intron	0.043 (T)
IKZF3	17	NM_012481	17:37944519	rs112301322	SNV	C/G	Exon	0.043 (G)
IKZF3	17	NM_012481	17:37948874	rs3803795	SNV	C/T	Intron	3.33200e-04 (T)

IKZF3	17	NM_012481	17:37985618	rs200283071	SNV	T/C	Intron	6.66134e-05 (T)
IKZF3	17	NM_012481	17:37985779	rs143669790	SNV	G/A	Intron	0.028 (A)
IKZF3	17	NM_012481	17:37985801	rs3816470	SNV	A/G	Intron	0.481 (A)
IKZF3	17	NM_012481	17:37988293	rs8069893	SNV	C/T	Intron	0.043 (T)
IKZF3	17	NM_012481	17:37988320	rs16965361	SNV	A/G	Intron	0.034 (G)
IKZF3	17	NM_012481	17:37988470	rs187289154	SNV	A/G	Intron	0.000 (G)
IKZF3	17	NM_012481	17:37988477	rs111944912	SNV	T/C	Intron	0.043 (C)
IKZF3	17	NM_012481	17:38020419	rs1453559	SNV	T/C	5'UTR	0.4919 (T)
IKZF3	17	NM_012481	17:38020421	rs117278702	SNV	G/A	5'UTR	0.039 (A)

SNV: Single Nucleotide Variant; INDEL: Insertion-Deletion Variant; UTR: Untranslated Region; NFE: Non-Finnish European

Table S3: List and characteristics of tools used in variants functional prediction

Predictor	Link	Type of Variant	Characteristics	Reference
Mutation Taster	http://mutationtaster.org	Any	Uses a Bayes classifier to generate predictions. Based on genomic databases, exon-intron junction and conservation analysis.	Schwarz JM, Rödelsperger C, Schuelke M, Seelow D. MutationTaster evaluates disease-causing potential of sequence alterations. *Nat Methods* 2010; **7**:575-576.
HSF	http://www.umd.be/HSF3/	Any	Predicts the effects of mutations on splicing signals. Identifies splicing motifs in any human sequence. It contains all available matrices for auxiliary sequence prediction.	Desmet FO, Hamroun D, Lalande M, Collod-Béroud G, Claustres M, Béroud C. Human Splicing Finder: an online bioinformatics tool to predict splicing signals. *Nucleic Acids Res* 2009; **37**:e67.
miRbase	http://www.mirbase.org	Any	It is a primary microRNA repository, using deep sequencing data sets. Allows to determine the miRNAs aligned in each sequence.	Kozomara A, Griffiths-Jones S. miRBase: annotating high confidence microRNAs using deep sequencing data. *Nucleic Acids Res* 2014; **42**:D68-73.
motifBreakR	https://bioconductor.org/packages/release/bioc/vignettes/motifbreakR/inst	Any	Works with position probability matrices (PPM), which contains information about the likelihood of observing a particular nucleotide at a particular position of a true transcription factor binding site.	Coetzee SG, Coetzee GA, Hazelett DJ. motifbreakR: an R/Bioconductor package for predicting variant effects at transcription factor

	/doc/motifbreakR-vignette.html			binding sites. *Bioinformatics* 2015; **31**:3847-3849.
CpG Island	http://www.urogene.org/cgi-bin/methprimer/methprimer.cgi	Any	Predicts CpG islands from an original DNA sequence and provides the virtual bisulfite conversion.	Li LC, Dahiya R. MethPrimer: designing primers for methylation PCRs. *Bioinformatics* 2002; **18**:1427-1431.
EnhancerAtlas	http://www.enhanceratlas.org	Any	Annotation database of enhancers in human and mouse genome. Examines the experimental evidences for enhancers in a given genomic region, compares the enhancers across different cells and tissues, identifies the enhancers associated with a given gene.	Gao T, He B, Liu S, Zhu H, Tan K, Qian J. EnhancerAtlas: a resource for enhancer annotation and analysis in 105 human cell/tissue types. *Bioinformatics* 2016; **32**:3543-3551.
SIFT	http://sift.jcvi.org	Missense	Uses an alignment based score to measure the change in sequence similarity, comparing to a protein homolog, before and after amino acid substitution.	Choi Y, Sims GE, Murphy S, Miller JR, Chan AP. Predicting the functional effect of amino acid substitutions and indels. *PLoS One* 2012; **7**:e46688.
PolyPhen-2	http://genetics.bwh.harvard.edu/pph2/	Missense	Uses eight sequence-based and three structure-based predictive features, selected by an algorithm, which compare the properties of the wild-type and mutated alleles.	Adzhubei I, Jordan DM, Sunyaev SR. Predicting functional effect of human missense mutations using PolyPhen-2. *Curr Protoc Hum Genet* 2013; **Chapter 7**:Unit7.20.

APASdb	http://mosas.sysu.edu.cn/utr	Any	Describes the polyadenylation consensus sequences, canonic and alternative ones, and describes which one is used in different tissues and transcripts.	You L, Wu J, Feng Y *et al.* APASdb: a database describing alternative poly(A) sites and selection of heterogeneous cleavage sites downstream of poly(A) signals. *Nucleic Acids Res* 2015; **43**:D59-67.
Haploview	https://www.broadinstitute.org/haploview/haploview	SNVs	Analyses and provides a graphic visualization of Linkage Disequillibrium (LD) between SNVs	Barrett JC, Fry B, Maller J, Daly MJ. Haploview: analysis and visualization of LD and haplotype maps. *Bioinformatics* 2005; **21**:263-265.
SILVA	http://compbio.cs.toronto.edu/silva/	Synonymous	Algorithm for priorization and identification of disease-causing synonymous variants	Buske OJ, Manickaraj A, Mital S, Ray PN, Brudno M. Identification of deleterious synonymous variants in human genomes. *Bioinformatics* 2013; **29**:1843-1850.
BiomaRt	https://bioconductor.org/packages/devel/bioc/vignettes/biomaRt/inst/doc/biomaRt.html	Any	R package developed by Ensembl database that combines data from different public repositories	Durinck S, Spellman PT, Birney E, Huber W. Mapping identifiers for the integration of genomic datasets with the R/Bioconductor package biomaRt. *Nat Protoc* 2009; **4**:1184-1191.
PredictSNP-2	https://loschmidt.chemi.muni.cz/predictsnp2/	SNVs	Combines data from six predictors: CADD, DANN, FATHMM, FitCons, FunSeq2 and GWAVA, and provides an integrated score of deleteriousness.	Bendl J, Musil M, Štourač J, Zendulka J, Damborský J, Brezovský J. PredictSNP2: A Unified Platform for Accurately Evaluating SNP Effects by Exploiting the Different Characteristics of Variants in Distinct

				Genomic Regions. *PLoS Comput Biol* 2016; **12**:e1004962.
RNA Fold	http://rna.tbi.univie.ac.at/	Exonic	Predicts messenger RNA (mRNA) secondary structure and free energy in its conformation.	Hofacker IL. Vienna RNA secondary structure server. *Nucleic Acids Res* 2003; **31**:3429-3431.

Table S4: Scores attributed to each functional prediction

Predictor	Impact	Score	Observation
Mutation Taster	Polymorphism	0	
	Splicing	+1	
	Protein features might be affected	+1	
	Disease causing	+3	
	Thalidomide site might be lost	+4	
HSF	No alteration of splicing	0	
	Alteration of ESSE/ESS	+1	for each site
	Activation exonic cryptic acceptor/donor site	+2	for each site
miRbase	miRNA non affected	0	
	miRNA non related affected	+1	
	miRNA related affected	+2	for each site
motifBreakR	No TF related affected	0	
	Alters TFs involved in embryogenesis	+1	for each site
	Alters TFs involved in limb development	+2	for each site
	Alters IKZF1, IKZF3 or MEIS2 binding site	+3	for each site
CpG Island	No CpG island in the region	0	
	Do not alter CpG Island	0	
	Alters beginning/end of CpG Island	+1	for each site
	Creates or disrupts CpG island	+3	for each site
EnhancerAtlas	Do not have enhancer	0	
	Inside enhancer of the gene	+1	for each site
	Disrupts enhancer of the gene	+3	for each site
SIFT<br>PolyPhen-2<br>SILVA	Tolerated, likely benign, neutral	0	
	Possibly Pathogenic	+2	
	Pathogenic	+4	
Síios de PoliA	Disrupts any polyA site or consensus sequence	+1	
	Disrupts main polyA site or consensus sequence	+2	
Haploview	It is located in haplotypic block	+1	
Fisher/Chi-Square	Allelic frequency after FDR <0.05	+1	
	Genotypic frequency after FDR<0.05	+1	
BiomaRt	Clinical significance	+5	
	MAF<0.01	+2	
	Splicing Site	+1	

	Exonic	+2
	New variant	+1
Predict-SNP2	Deleterious	+1
	DANN Deleterious	+1
RNA Fold	Alters mRNA stability	+1
	Alters mRNA conformation	+1
	Disrupts mRNA conformation	+2

Table S5: Association between upper limbs anomalies and variants identified

All Genes					
**Type of Anomaly**	**N**	**Mean Number of Variants**	**P-Value***	**Mean Heatmap Score**	**P-Value***
Intercalary transverse	14	32.43 (±6.02)	0.141	195 (±41.75)	0.048
Pre-axial longitudinal	10	36.70 (±7.12)		228.9 (±34.9)	

*t-Test

CRBN					
**Type of Anomaly**	**N**	**Mean Number of Variants**	**P-Value***	**Mean Heatmap Score**	**P-Value***
Intercalary transverse	14	10.85 (± 2.44)	0.241	78 (± 18.37)	0.016
Pre-axial longitudinal	10	11.9 (± 1.79)		94.9 (± 13.2)	

*t-Test

Table S6: Comparison of the presence of variants in Thalidomide Embryopathy Individuals and Europeans from the 1000Genomes project

All Genes					
**Groups**	**N**	**Mean Number of Variants**	**P-Value***	**Mean Heatmap Score**	**P-Value***
Thalidomide Embryopathy	35	36.17 (± 7.19)	<0.001	222.17 (± 44.84)	<0.001
Europeans 1000Genomes	99	29.96 (± 7.47)		179.39 (± 42.65)	

*t-Test

CRL4 (*CRBN + DDB1 + CUL4A)*					
**Type of Anomaly**	**N**	**Mean Number of Variants**	**P-Value***	**Mean Heatmap Score**	**P-Value***
Thalidomide Embryopathy	35	22.51 (±4.57)	<0.001	159.17 (± 35.55)	0.001
Europeans 1000Genomes	99	18.8 (± 3.31)		134.61 (±28.66)	

*t-Test

CRBN					
**Type of Anomaly**	**N**	**Mean Number of Variants**	**P-Value***	**Mean Heatmap Score**	**P-Value***
Thalidomide Embryopathy	35	11.71 (± 2.24)	0.001	87.94 (± 17.88)	0.038
Europeans 1000Genomes	99	10.21 (± 1.83)		80.60 (± 16.52)	

*t-Test

DDB1					
**Type of Anomaly**	**N**	**Mean Number of Variants**	**P-Value***	**Mean Heatmap Score**	**P-Value***
Thalidomide Embryopathy	35	1.97 (± 2.71)	0.001	14.37 (± 19.34)	<0.001
Europeans 1000Genomes	99	0.22 (± 0.44)		1.43 (± 3.01)	

*t-Test

CUL4A					
**Type of Anomaly**	**N**	**Mean Number of Variants**	**P-Value***	**Mean Heatmap Score**	**P-Value***
Thalidomide Embryopathy	35	8.86 (± 2.71)	0.362	56.86 (± 22.78)	0.346
Europeans 1000Genomes	99	8.36 (± 2.79)		52.57 (± 22.33)	

*t-Test

IKZF1					
**Type of Anomaly**	**N**	**Mean Number of Variants**	**P-Value***	**Mean Heatmap Score**	**P-Value***
Thalidomide Embryopathy	35	9.51 (± 6.47)	0.073	49.8 (± 31.32)	0.006
Europeans 1000Genomes	99	7.23 (± 6.00)		32.6 (± 27.14)	

*t-Test

IKZF3					
**Type of Anomaly**	**N**	**Mean Number of Variants**	**P-Value***	**Mean Heatmap Score**	**P-Value***
Thalidomide Embryopathy	35	4.14 (± 2.04)	0.609	13.2 (± 9.15)	0.575
Europeans 1000Genomes	99	3.94 (± 1.9)		12.18 (± 9.27)	

*t-Test

Table S7: ANOVA test between individuals with pre-axial longitudinal limb anomalies vs. Intercalary transverse limb anomalies vs. Europeans from the 1000Genomes project

Presence of Variants (Absolute Number)		
Genes	F	P-Value
CRBN + DDB1 + CUL4A + IKZF1 + IKZF3	4.252	0.016
CRBN + DDB1 + CUL4A	6.717	0.002
CRBN	3.961	0.022
DDB1	16.124	<0.001
CUL4A	0.116	0.891
IKZF1	0.784	0.459
IKZF3	0.397	0.673

Heatmap Score		
Genes	F	P-Value
CRBN + DDB1 + CUL4A + IKZF1 + IKZF3	6.731	0.002
CRBN + DDB1 + CUL4A	4.799	0.01
CRBN	3.753	0.026
DDB1	16.609	<0.001
CUL4A	0.122	0.885
IKZF1	1.672	0.192
IKZF3	0.751	0.474

Table S8: Association between rs1045433 genotypes and patten of upper limb anomalies in TE individuals

CRBN - rs1045433			
**Genotypes**	**Intercalary Transverse n (%)**	**Pre-Axial Longitudinal n (%)**	**P-Value***
TT	13 (92.9%)	4 (40%)	0.004
TC	1 (7.1%)	2 (20%)	
CC	0 (0%)	4 (40%)	

*Fisher's Exact Test

Table S9. Differential Gene Expression of CRL4-CRBN Genes in Embryonal Human First to Fifth Digits

GENE	logFC	logCPM	LR	P-Value	FDR
CRBN	0.171523	5.979662	0.471413	0.492338	1
CUL4A	-0.02522	8.668676	0.010878	0.916933	1
DDB1	0.00751	6.721533	0.001085	0.973727	1
RBX1	0.131598	5.346193	0.21163	0.645493	1
IKZF1	-0.3733	1.424598	0.391919	0.531292	1
IKZF3	0	-1.88007	0	1	1

FDR: fale discovery rate P-adjusted value

Table S10. Evolutionary rates and likelihood ratio test for six genes integrating the CRBN complex

GENE	Models M1a *vs.* M2a			M8a *vs.* M8		
	ω	Proportion (p)	***LRT***	ω	Proportion (p)	***LRT***
CRBN	< 0.03061	0.89589	*p* > 0.99	< 0.06875	0.858	*p* > 0.99
CUL4A	< 0.01869	0.97541	*p* > 0.99	< 0.01869	0.897	*p* > 0.71
DDB1	< 0.00625	0.98907	*p* > 0.99	< 0.06770	0.999	*p* > 0.88
RBX1	< 0.00233	0.999	*p* > 0.99	< 0.01265	0.999	*p* > 0.99
IKZF1	< 0.04991	0.92174	*p* > 0.99	< 0.06734	0.7	*p* > 0.99
IKZF3	< 0.05351	0.89144	*p* > 0.99	< 0.08374	0.778	*p* > 0.99

*Degrees of freedom: 2;

**The omega values and proportions refer to neutral models (M1a and M8a).

Table S11: Differential Gene Expression of CRL4-CRBN Genes in Mouse Embryonic Stem Cells (mESC) after Thalidomide Exposure

24 hours			
Genes	Relative Gene Expression		P-Value
	Saline (n=3)	Thalidomide (n=3)	
Crbn	8.27009	7.916073	0.0759
Ddb1	10.49194	10.63323	0.0721
Cul4a	8.365498	8.043906	**0.0068**
Rbx1	9.610183	9.382166	0.0635
Ikzf1	5.114148	5.095873	0.6359
Ikzf3	4.790702	5.112851	0.2

48 hours			
Genes	Relative Gene Expression		P-Value
	Saline (n=3)	Thalidomide (n=3)	
Crbn	8.236536	7.879079	**0.0244**
Ddb1	10.6935	10.84967	**0.0365**
Cul4a	8.26444	7.942627	**0.0195**
Rbx1	9.580732	9.28862	**0.0117**
Ikzf1	4.27323	4.375998	0.3252
Ikzf3	4.439432	4.528063	0.3261

72 hours			
Genes	Relative Gene Expression		P-Value
	Saline (n=3)	Thalidomide (n=3)	
Crbn	8.444035	8.342036	0.217
Ddb1	10.92195	10.9002	0.4821
Cul4a	8.641674	8.552191	0.0781
Rbx1	9.90335	9.857822	0.5563
Ikzf1	4.534075	4.768691	0.256
Ikzf3	4.79187	4.917498	0.3067

Table S12: Differential Gene Expression of CRL4-CRBN Genes in Monkey Embryos after Thalidomide Exposure

6 hours			
Genes	Relative Gene Expression		P-Value
	Control (n=2)	Thalidomide (n=3)	
Crbn	7.968	7.76	0.3532
Ddb1	9.253	9.403	0.4
Cul4a	7.302	6.529	**0.0121**
Rbx1	10.924	10.9	1
Ikzf1	3.302	3.331	0.9024
Ikzf3	3.781	3.752	1

Table S13. Differential Gene Expression of CRL4-CRBN Genes in Human Progenitor Stem Cells, 2 and 6 Days after Exposure

GENE	2 Days After Exposure					6 Days After Exposure				
	logFC	logCPM	LR	P-Value	FDR	logFC	logCPM	LR	P-Value	FDR
CRBN	0.181874	5.644369	1.351001	0.245103	0.707643	0.181874	5.644369	1.351001	0.245103	0.707643
CUL4A	-0.03389	8.286705	0.122679	0.726147	1	-0.03389	8.286705	0.122679	0.726147	1
DDB1	0.092972	6.692616	0.459681	0.497773	0.973657	0.092972	6.692616	0.459681	0.497773	0.973657
RBX1	-0.18149	5.930839	2.033754	0.15384	0.583658	-0.18149	5.930839	2.033754	0.15384	0.583658
IKZF1	-0.82007	1.202056	1.78253	0.181839	0.642126	-0.82007	1.202056	1.78253	0.181839	0.642126
IKZF3	0	-1.20123	0	1	1	0	-1.20123	0	1	1

FDR: fale discovery rate P-adjusted value

Supplementary Figures 1-14

CRL4-CEREBLON COMPLEX IN THALIDOMIDE EMBRYOPATHY: A TRANSLATIONAL INVESTIGATION

Thayne Woycinck Kowalski

Julia do Amaral Gomes

Gabriela Barreto Caldas Garcia

Lucas Rosa Fraga

Vanessa Rodrigues Paixao-Cortes

Mariana Recamonde-Mendoza

Maria Teresa Vieira Sanseverino

Lavinia Schuler-Faccini

Fernanda Sales Luiz Vianna

Figure S1: *CUL4A* variants in the TE subjects

Number of variants in the *CUL4A* gene in each subject with TE, and their respective location in the gene; Genotypes of the *CUL4A* variants in each subject.

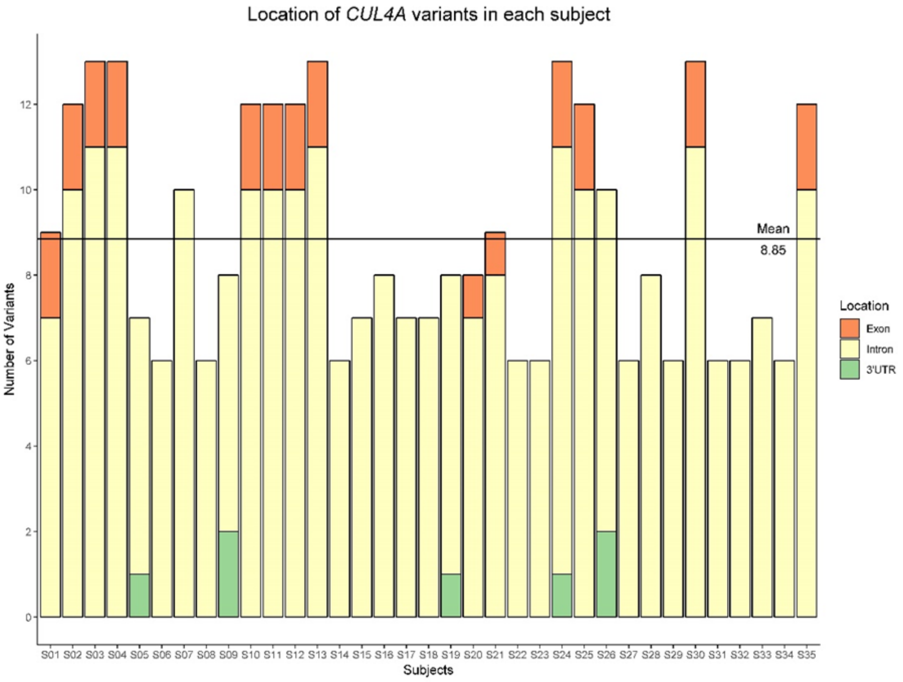

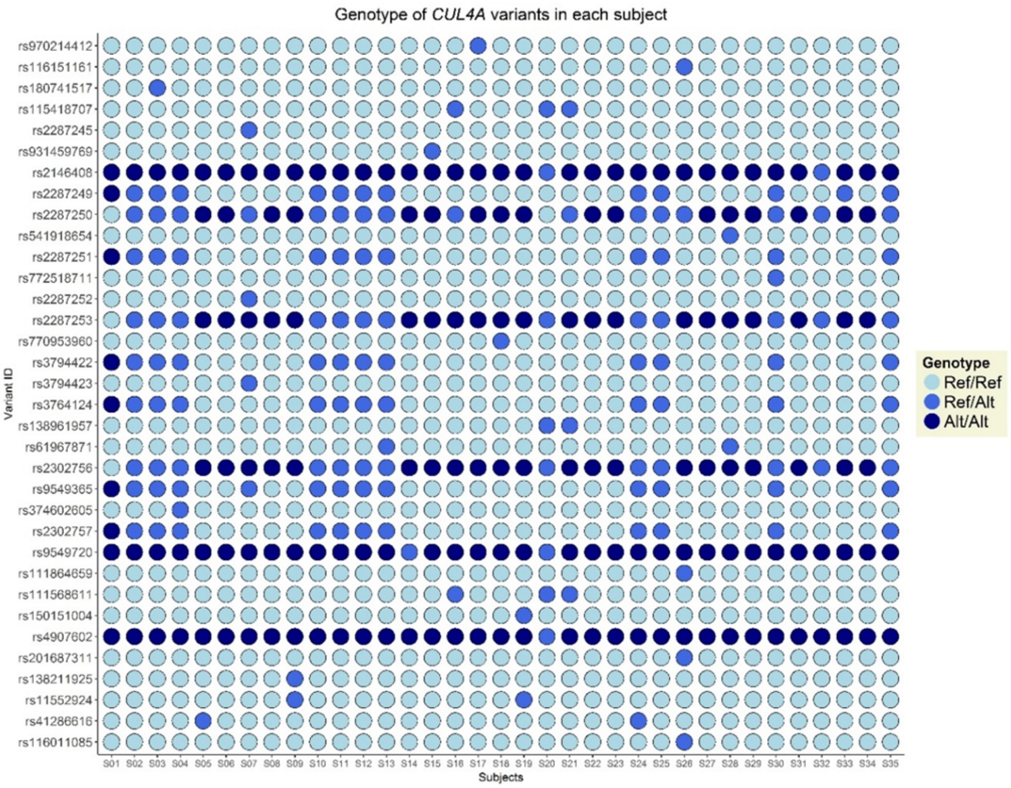

Figure S2: *DDB1* variants in the TE subjects

Number of variants in the *DDB1* gene in each subject with TE, and their respective location in the gene; Genotypes of the *DDB1* variants in each subject.

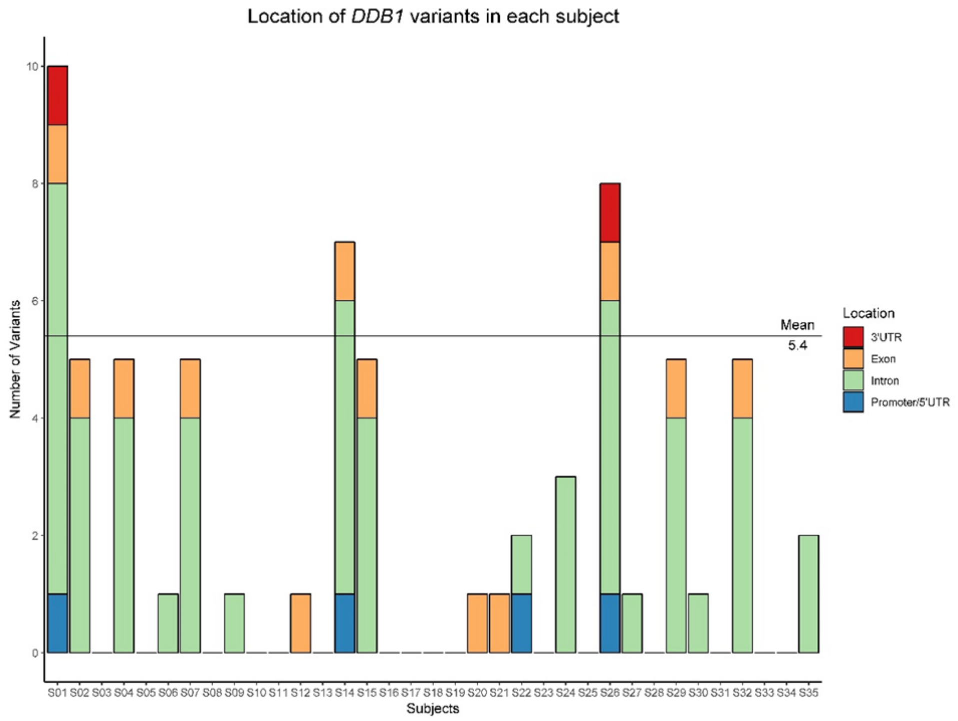

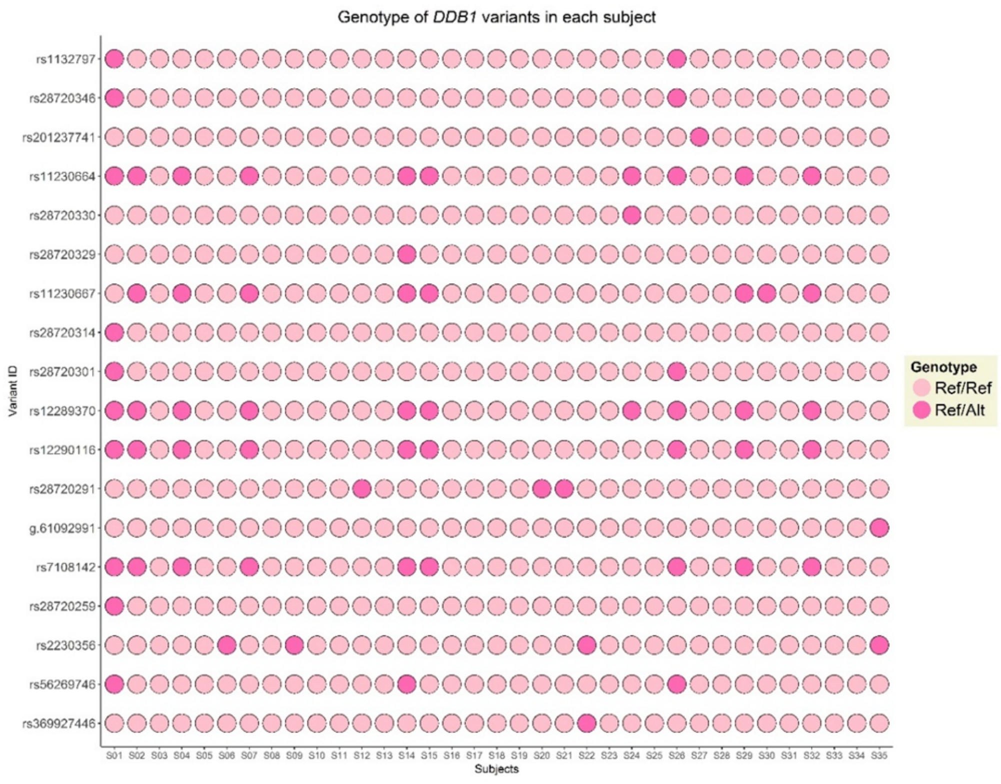

Figure S3: *IKZF1* variants in the TE subjects

Number of variants in the *IKZF1* gene in each subject with TE, and their respective location in the gene; Genotypes of the *IKZF1* variants in each subject.

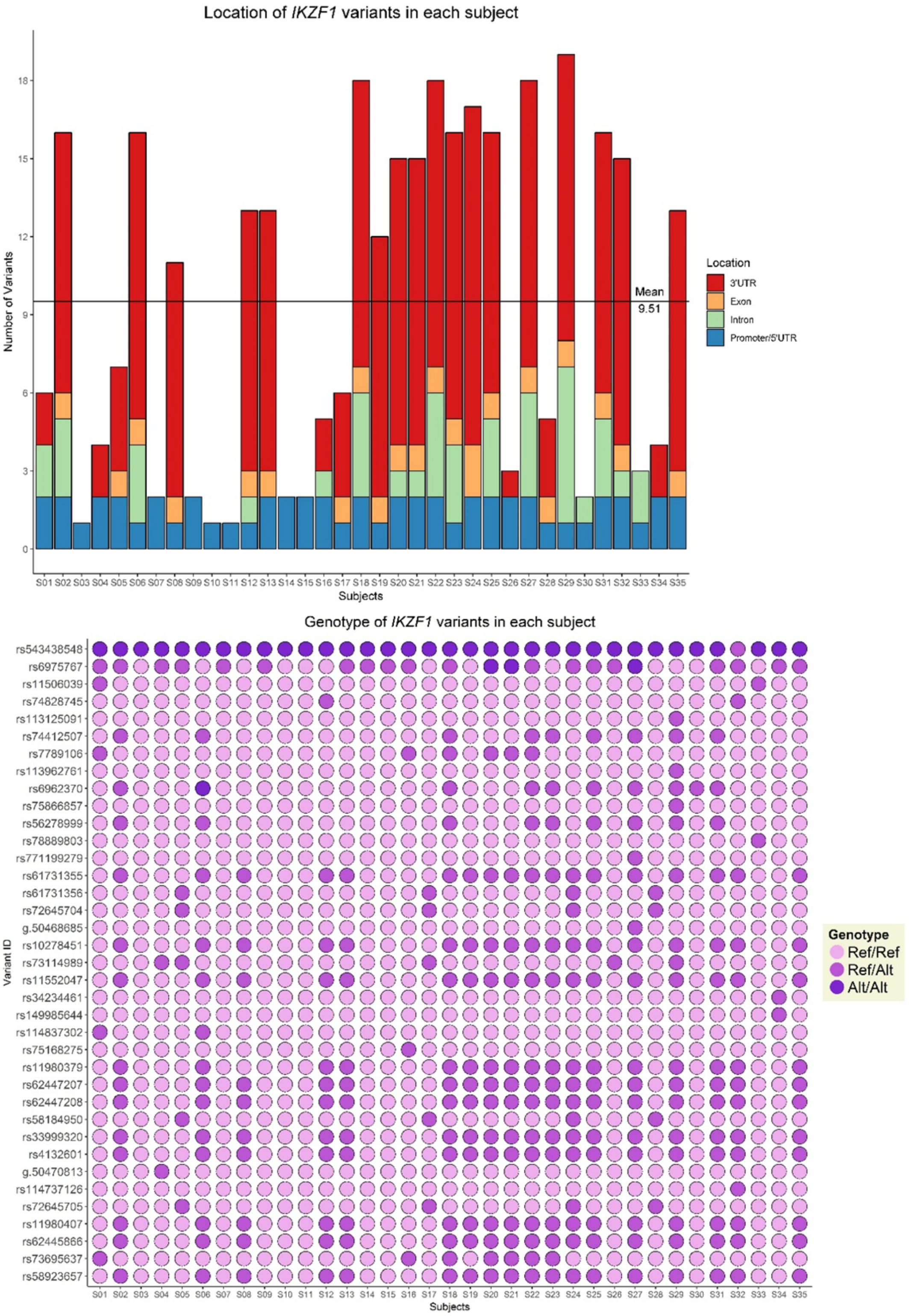

Figure S4: *IKZF3* variants in the TE subjects

Number of variants in the *IKZF3* gene in each subject with TE, and their respective location in the gene; Genotypes of the *IKZF3* variants in each subject.

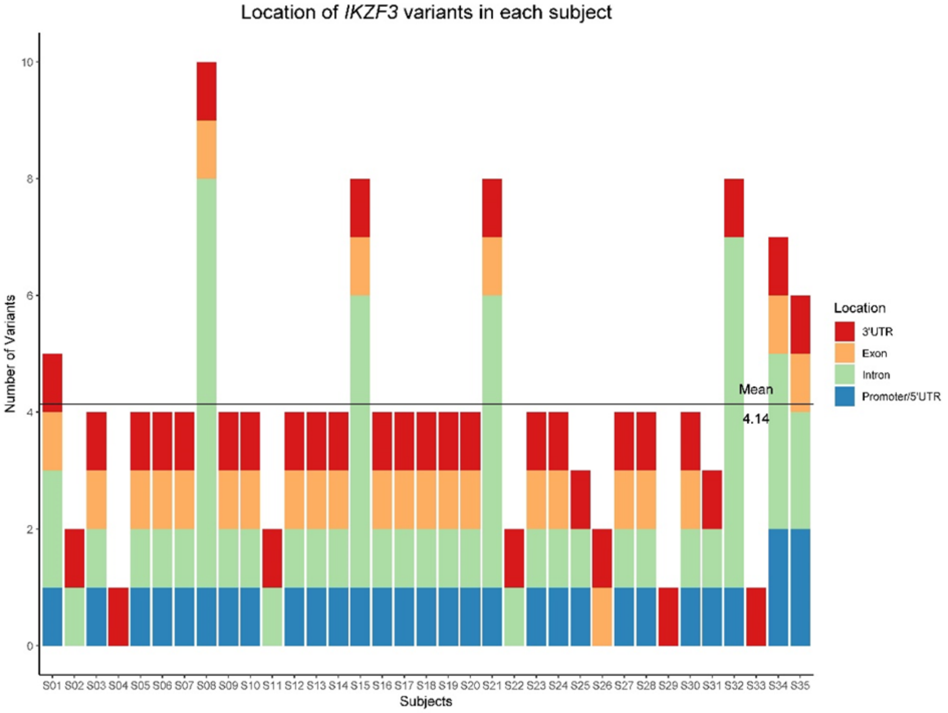

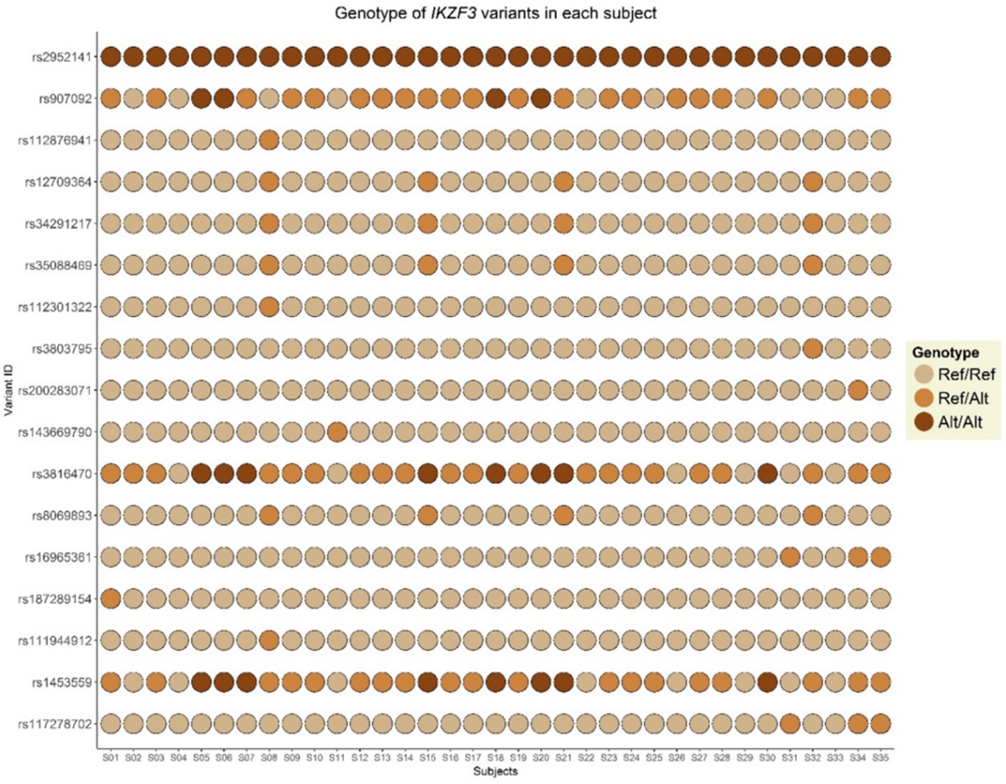

Figure S5: Heatmap of *CRBN* variants

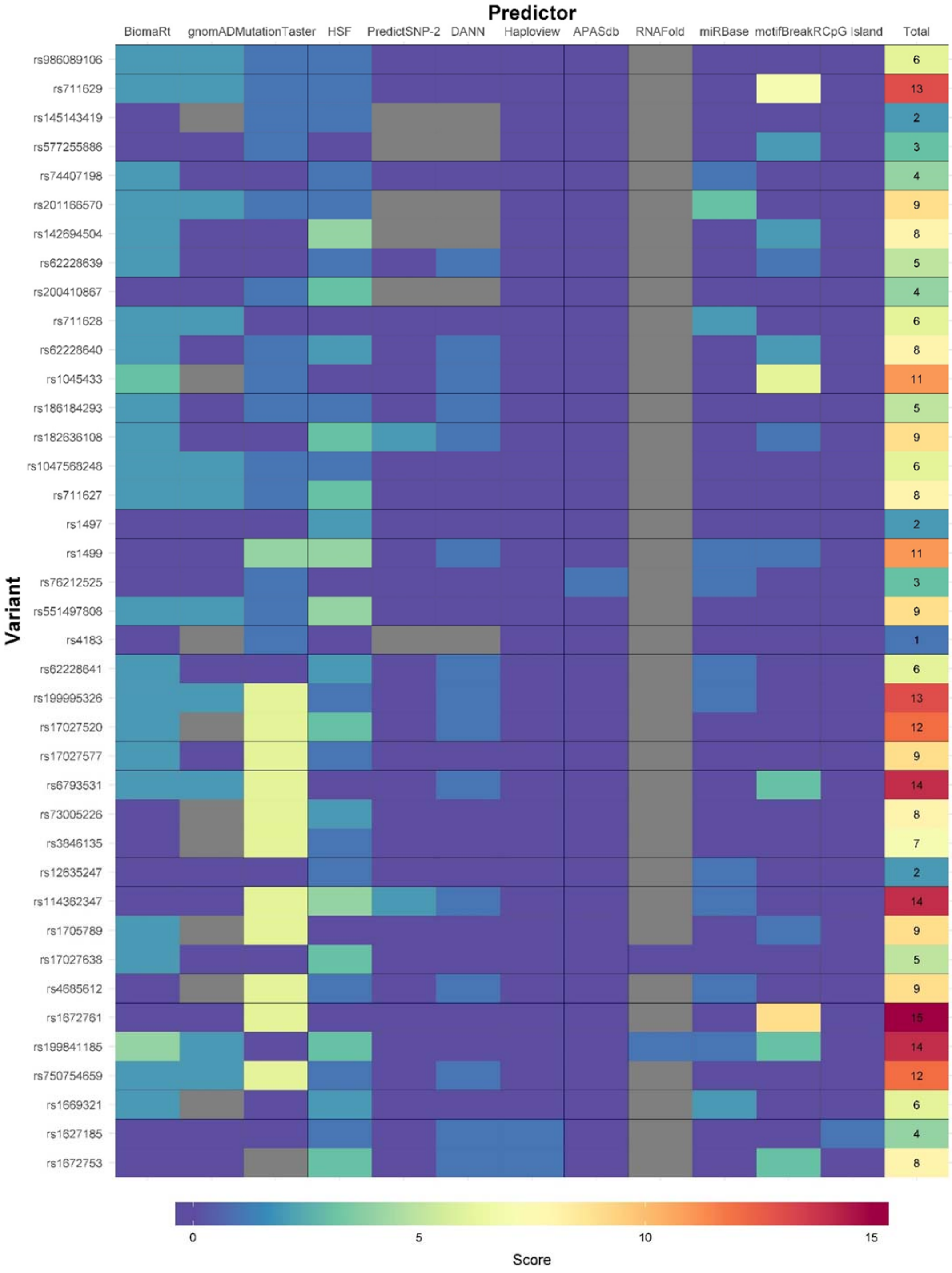

Figure S6: Heatmap of *CUL4A* variants

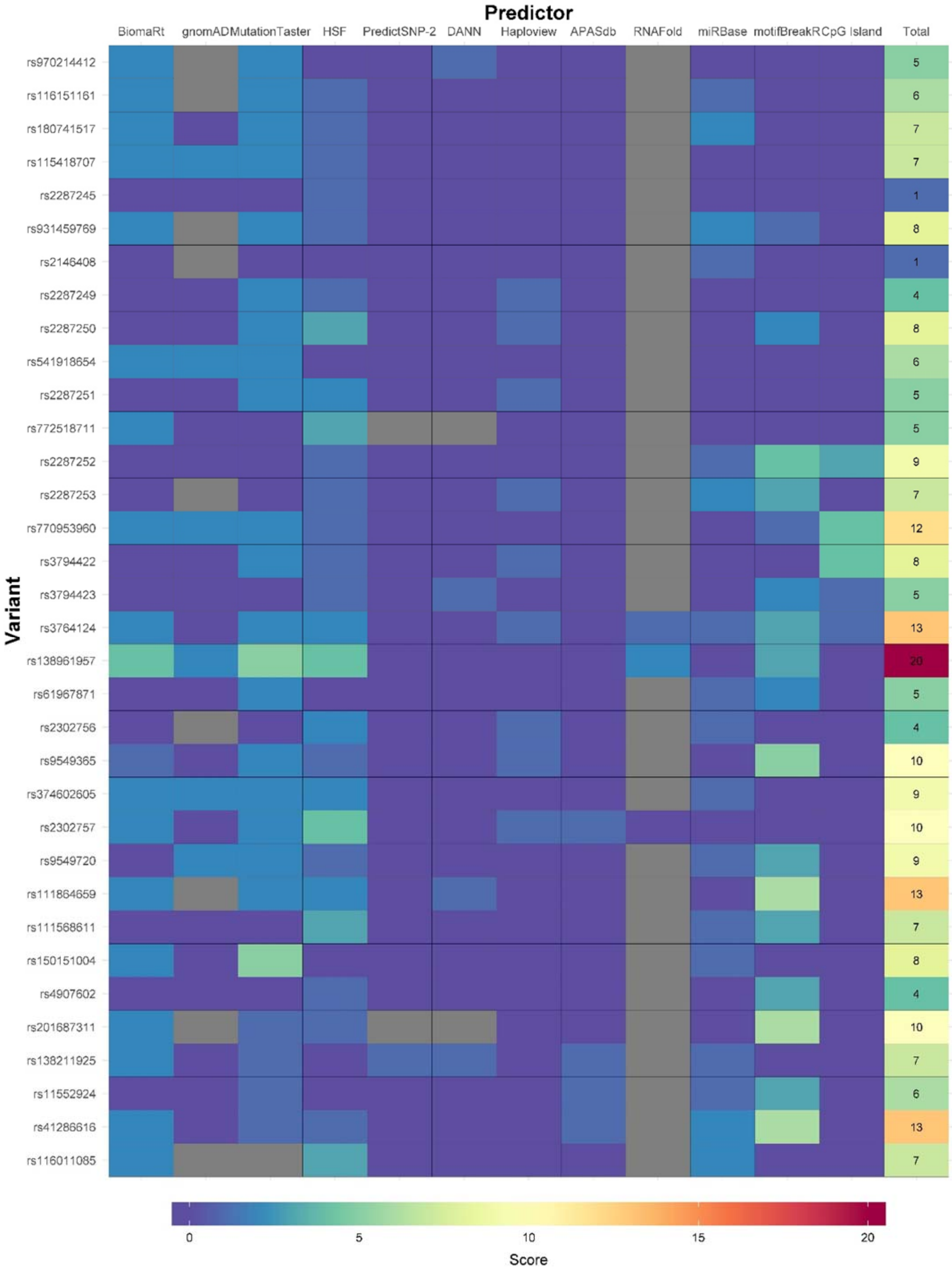

Figure S7: Heatmap of *DDB1* variants

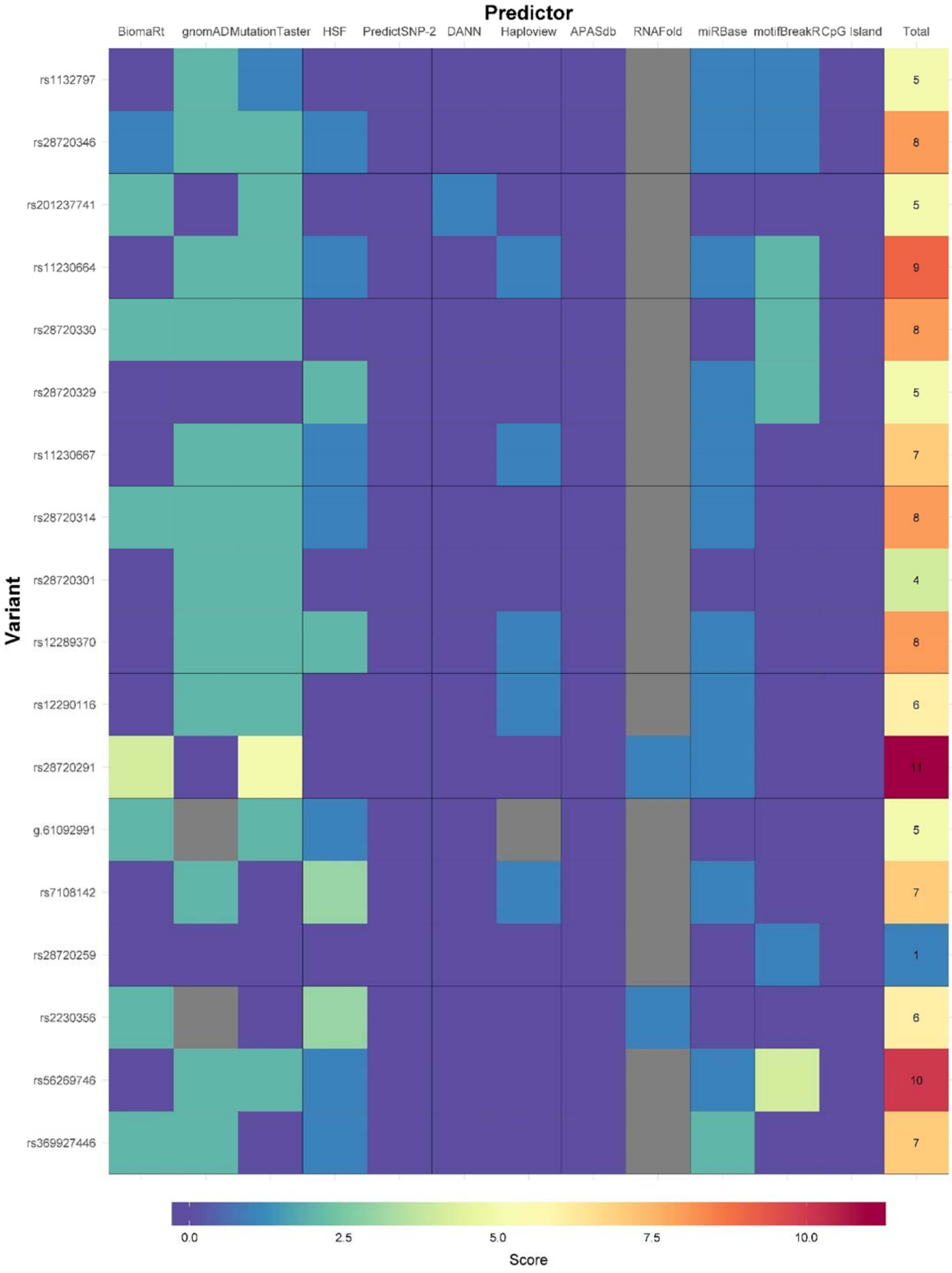

Figure S8: Heatmap of *IKZF1* variants

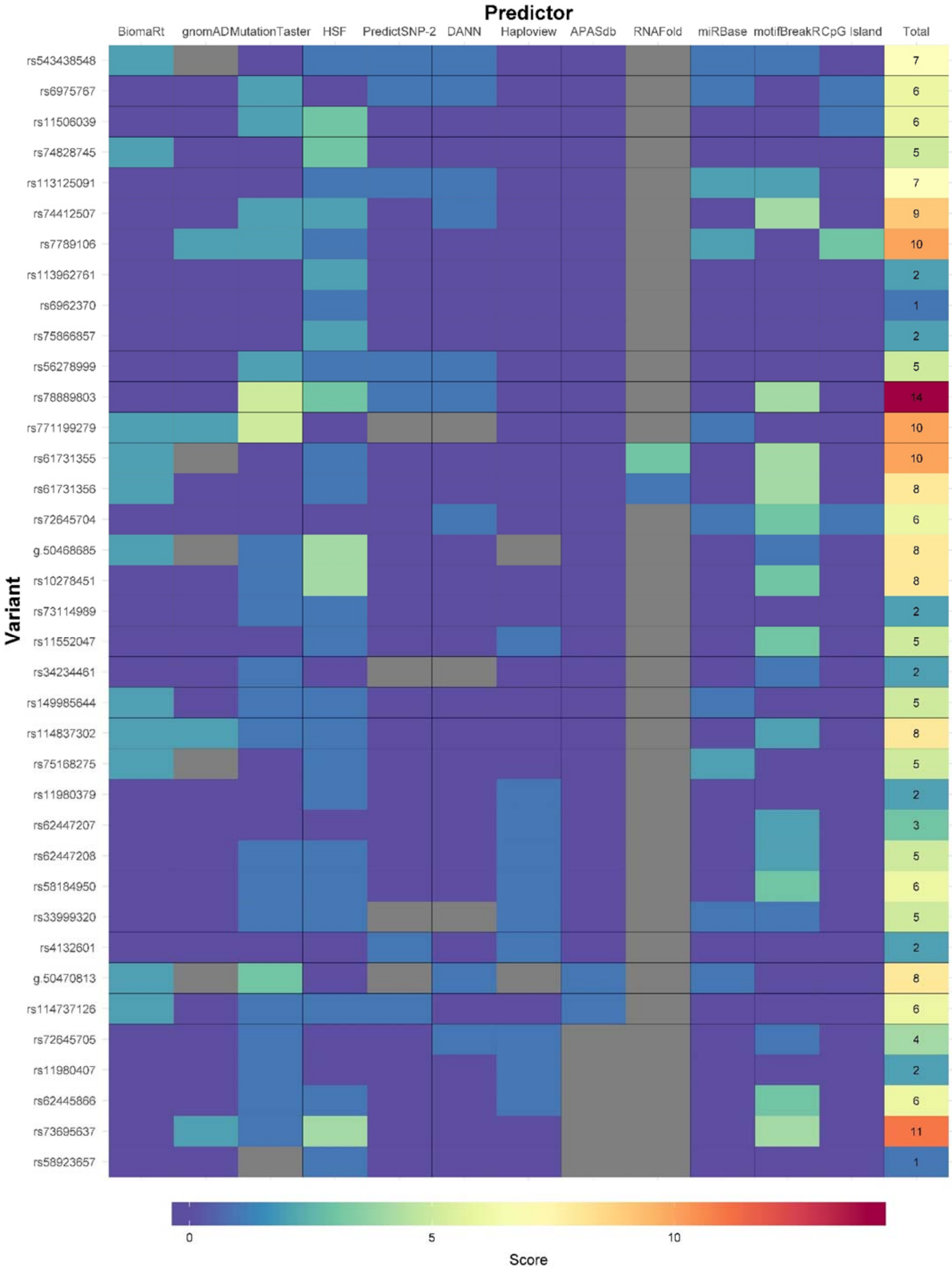

Figure S9: Heatmap of *IKZF3* variants

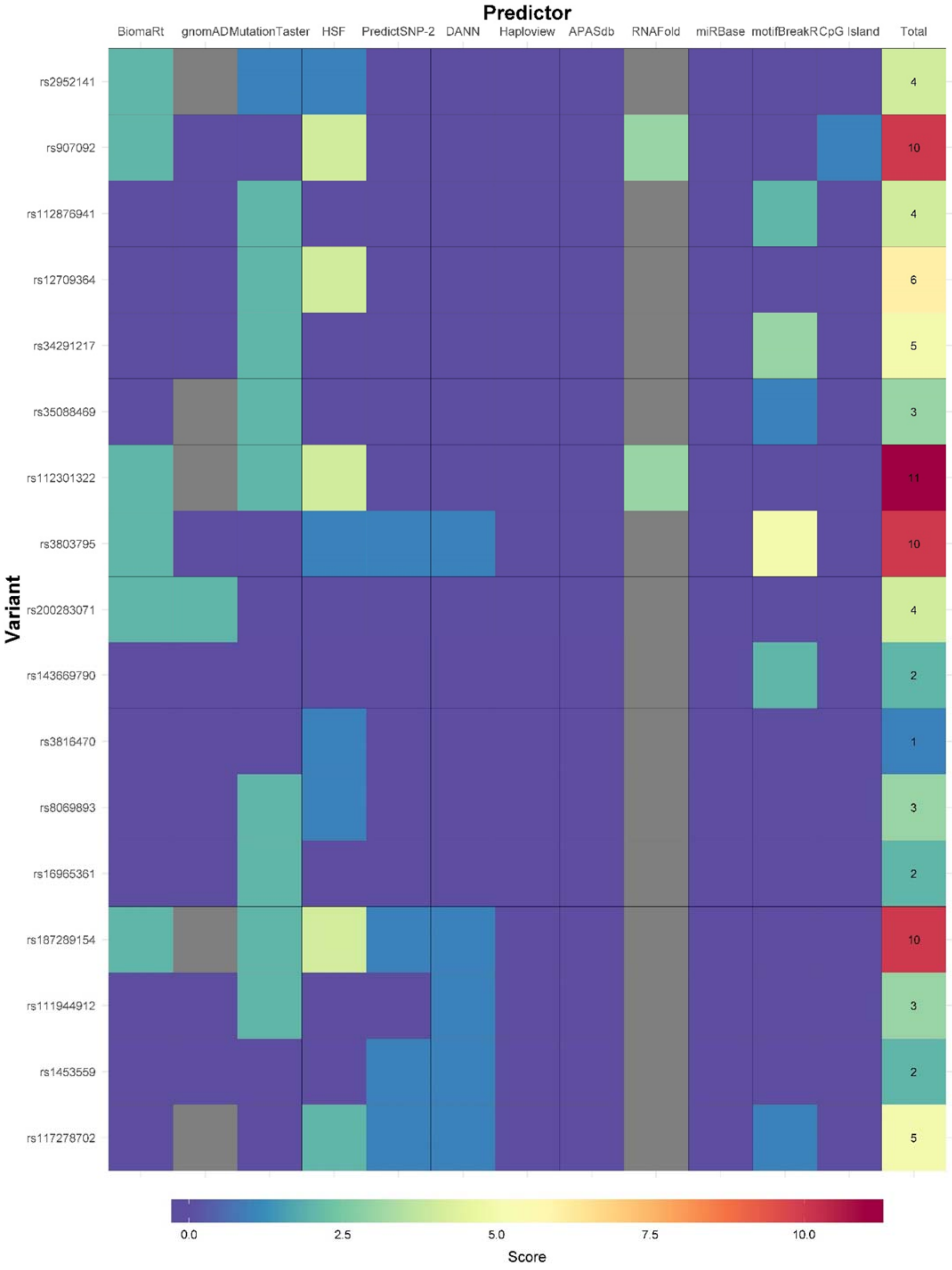

Figure S10: Structural changes in *CUL4A* and *IKZF1* mRNAs because of rs138961957 and rs61731355 synonymous variants, respectively.

CUL4A

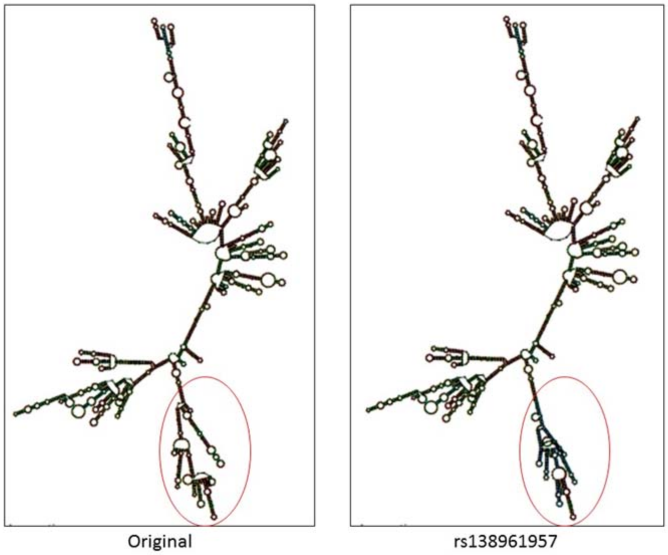

IKZF1

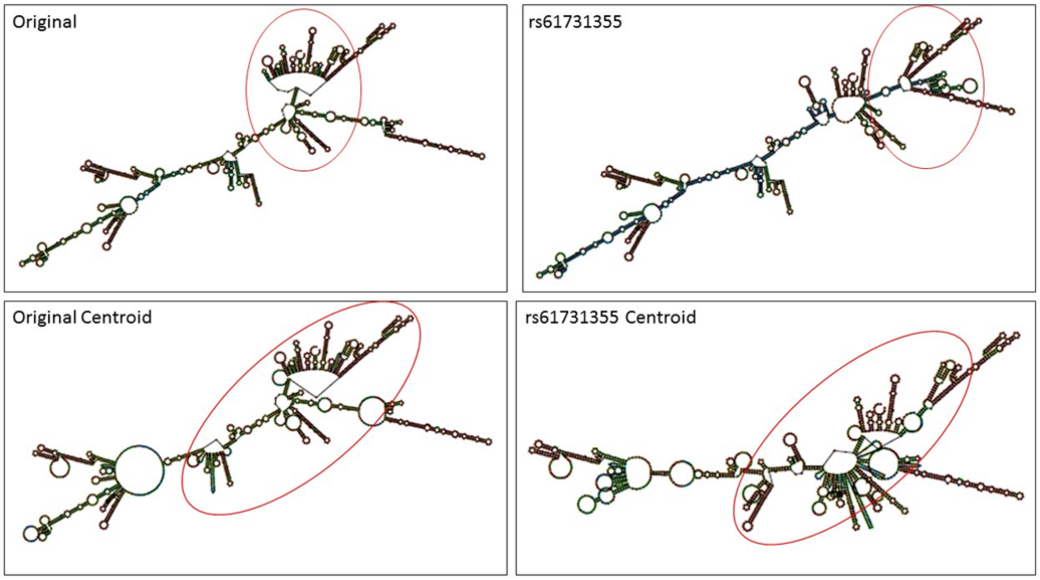

Figure S11: Representation of the location of each variant identified in the gene panel

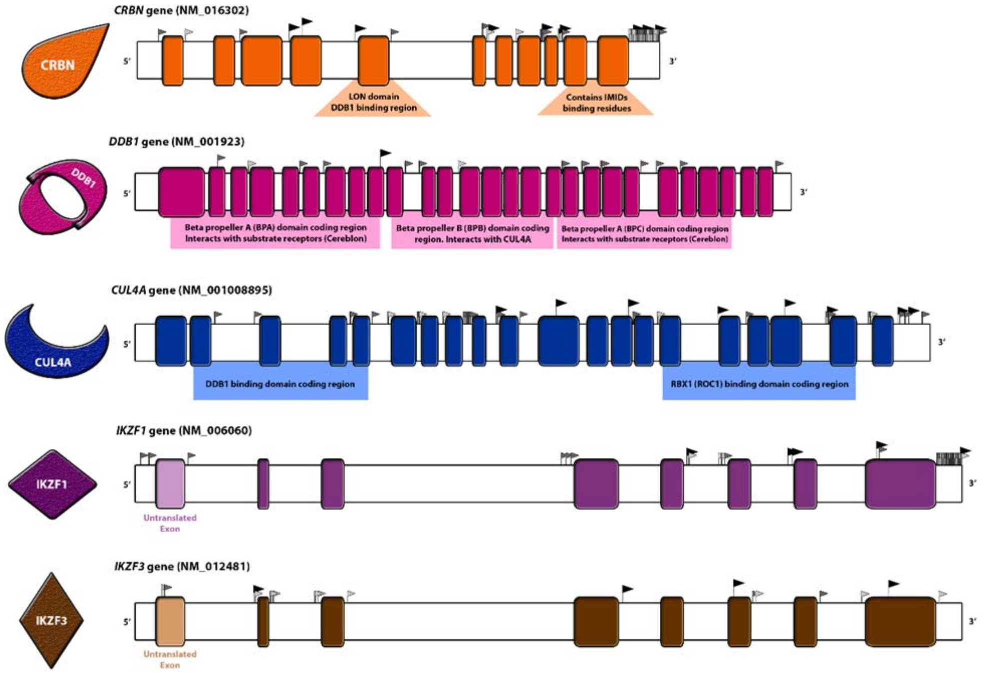

Flags represent the location of each variant. Light gray flags: variants of low score. Medium gray flags: variants of medium score. Black flags: variants of high score.

Figure S12: Gene expression in transcripts per million for limb development, CRL4^{CRBN} complex and reference genes.

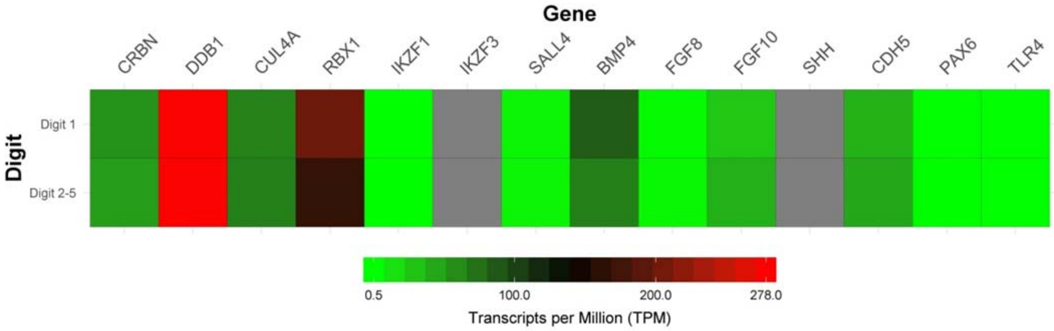

Legend: green colors: low expression; black: medium expression; red: high expression. Genes evaluated: *CRBN, DDB1, CUL4A, RBX1, IKZF1, IKZF3, SALL4, BMP4, FGF8, FGF10, SHH, CDH5, PAX6,* and *TLR4.*

Figure S13: *CRBN* gene methylation analyses in the TE sample

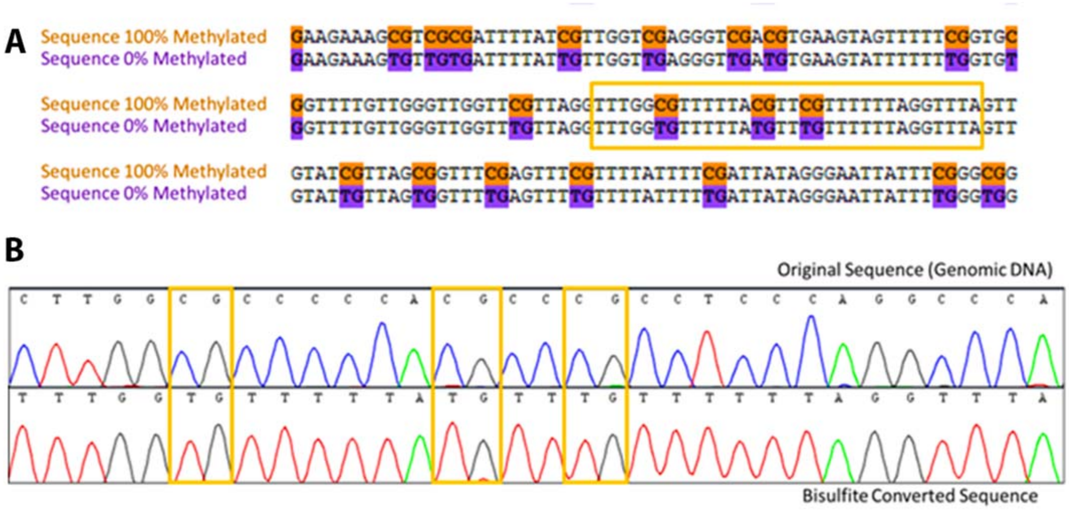

(A) DNA sequence after bisulfite conversion. Orange: nucleotides that would be encountered in total methylation. Purple: nucleotides that would be encountered in the absence of methylation. (B) Sanger sequencing. Yellow boxes mark CpG dinucleotides, with no methylation.

Figure S14: Pathways enriched in the CRL4 complex interacting proteins

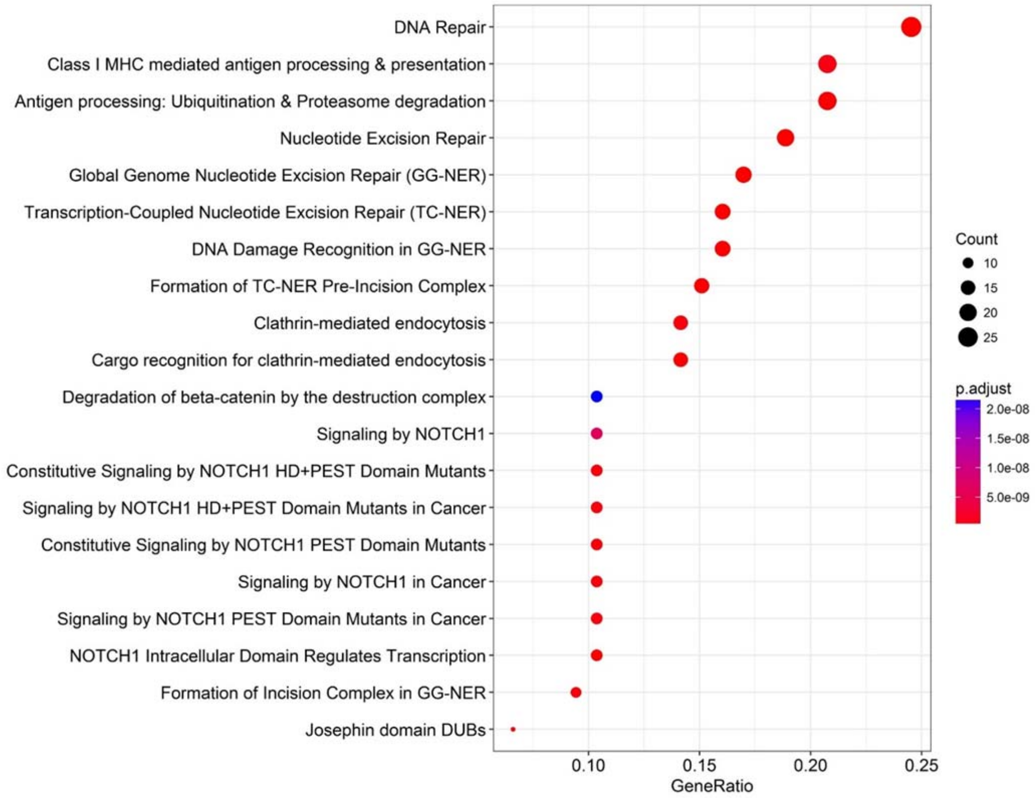

Supplementary Files 1 and 2

CRL4-CEREBLON COMPLEX IN THALIDOMIDE EMBRYOPATHY: A TRANSLATIONAL INVESTIGATION

Thayne Woycinck Kowalski

Julia do Amaral Gomes

Gabriela Barreto Caldas Garcia

Lucas Rosa Fraga

Vanessa Rodrigues Paixao-Cortes

Mariana Recamonde-Mendoza

Maria Teresa Vieira Sanseverino

Lavinia Schuler-Faccini

Fernanda Sales Luiz Vianna

Supplementary File

Alignment of *CRBN, DDB1* and *CUL4A* genes and proteins in eight species

Source: UNIPROT

CRBN

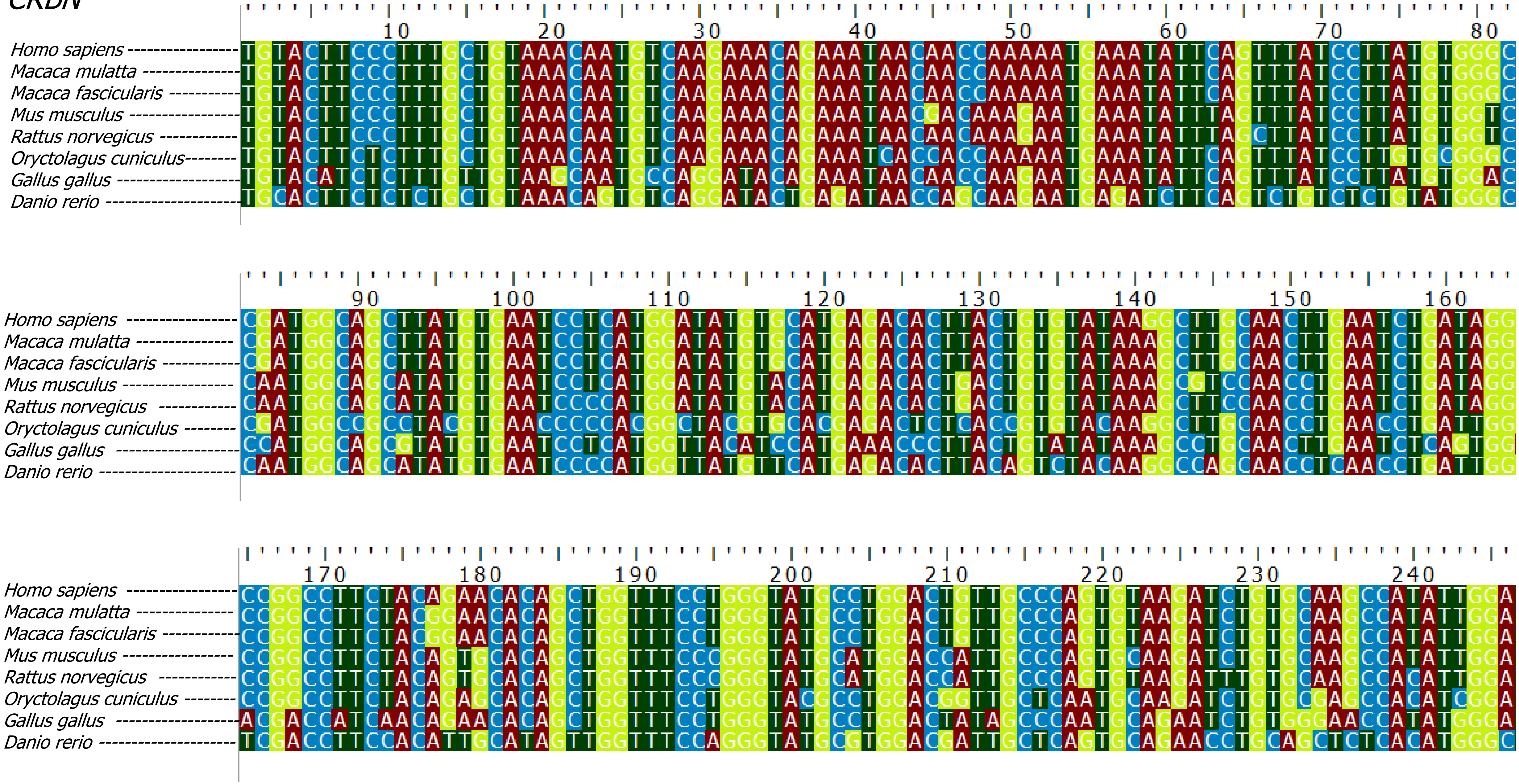
Homo sapiens
Macaca mulatta
Macaca fascicularis
Mus musculus
Rattus norvegicus
Oryctolagus cuniculus
Gallus gallus
Danio rerio

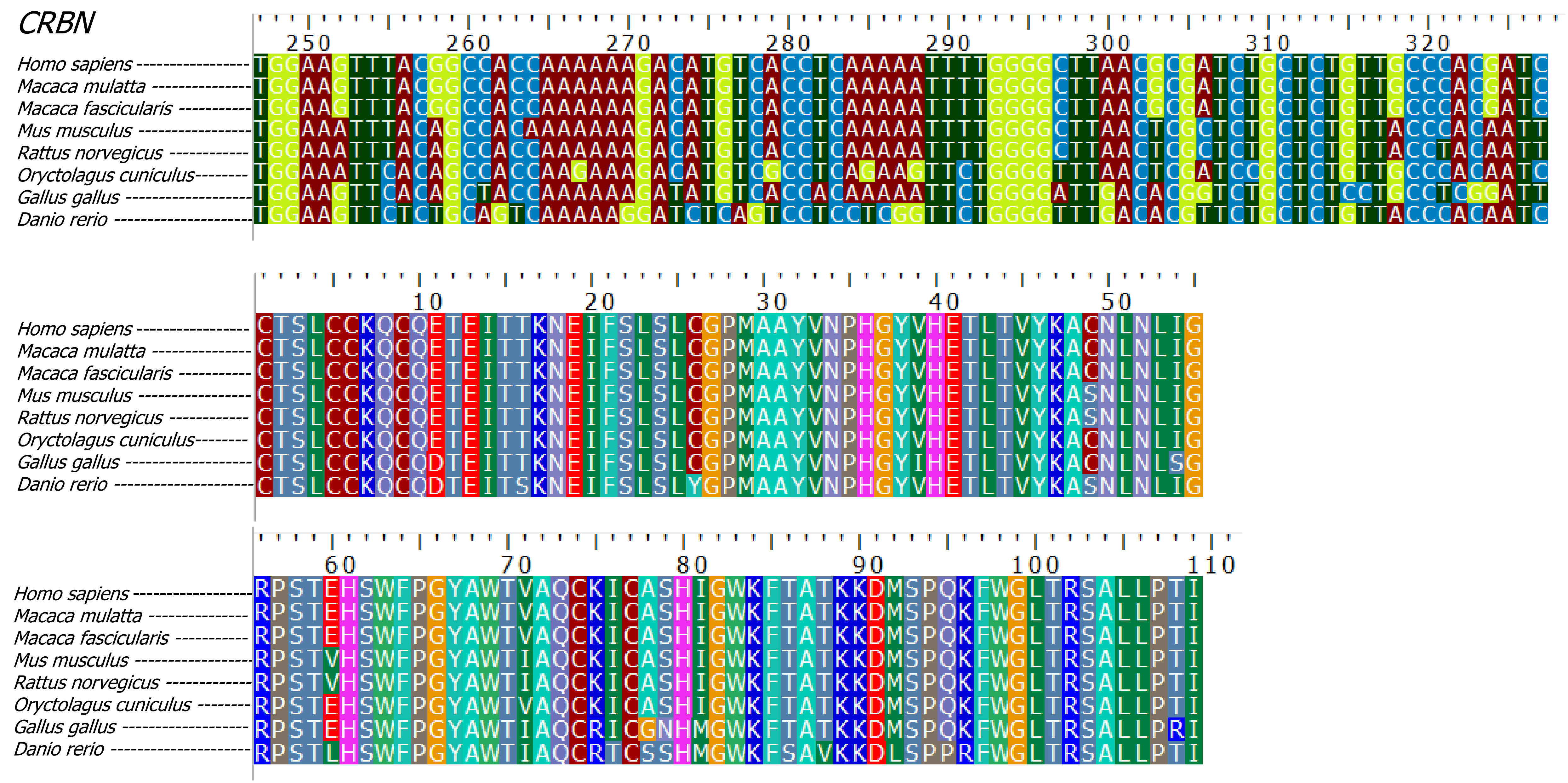

CRBN gene - the well conserved CULT domain (318-426), DNA and protein sequences.
*Source: UniProt

DDB1

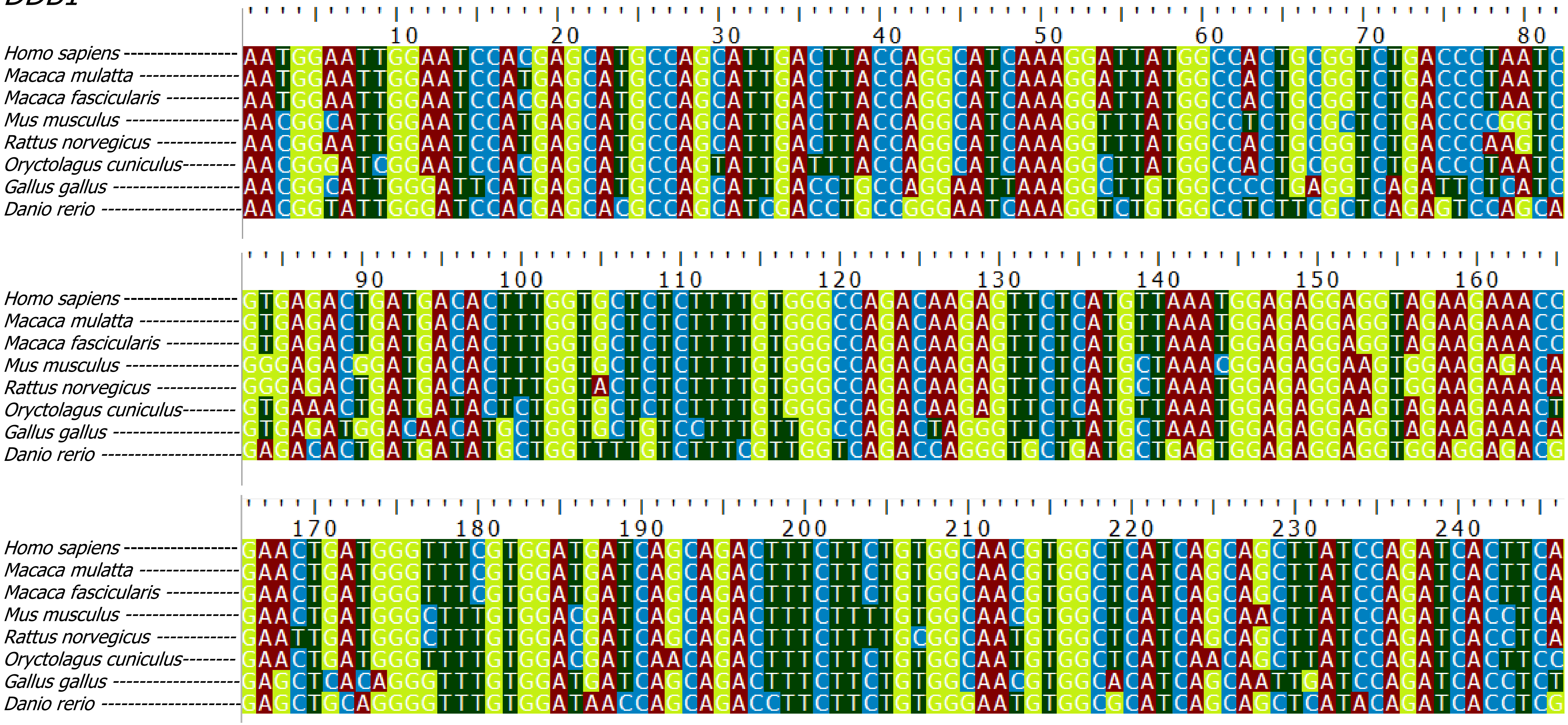

Homo sapiens
Macaca mulatta
Macaca fascicularis
Mus musculus
Rattus norvegicus
Oryctolagus cuniculus
Gallus gallus
Danio rerio
10 20 30 40 50 60 70 80
90 100 110 120 130 140 150 160
170 180 190 200 210 220 230 240

DDB1

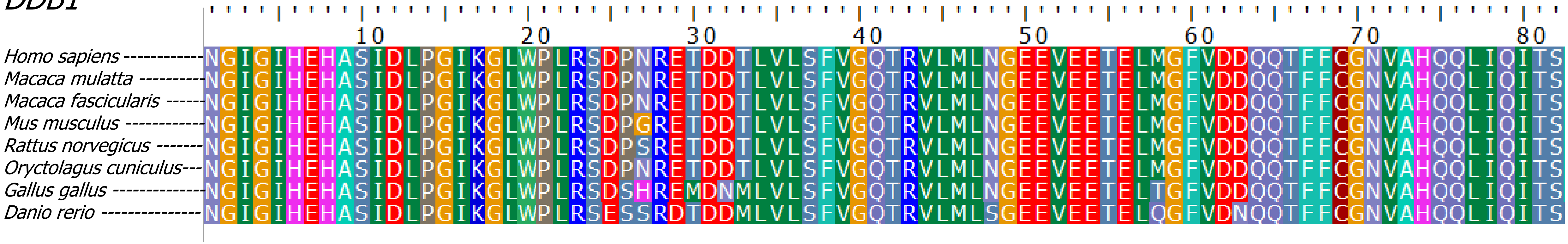

DDB1 gene*:* first 246 nucleotides and their 82 corresponding amino acids from the WD repeat beta-propeller B; Interaction with CUL4A region. Only some of the amino acids (392-473) are shown. The full region has 317 amino acids (392-708).
*Source: UniProt

CUL4A

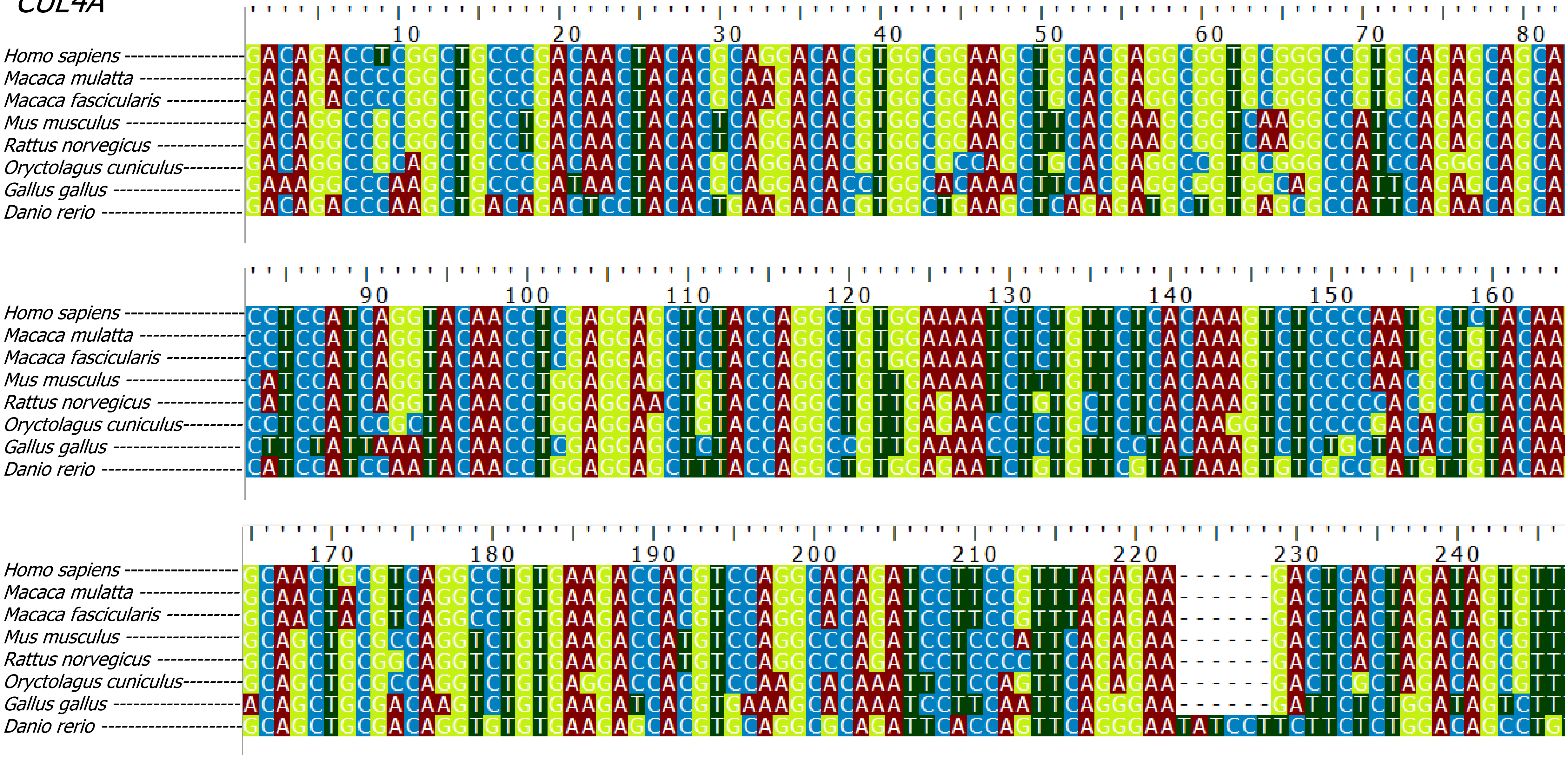
Homo sapiens
Macaca mulatta
Macaca fascicularis
Mus musculus
Rattus norvegicus
Oryctolagus cuniculus
Gallus gallus
Danio rerio

CUL4A

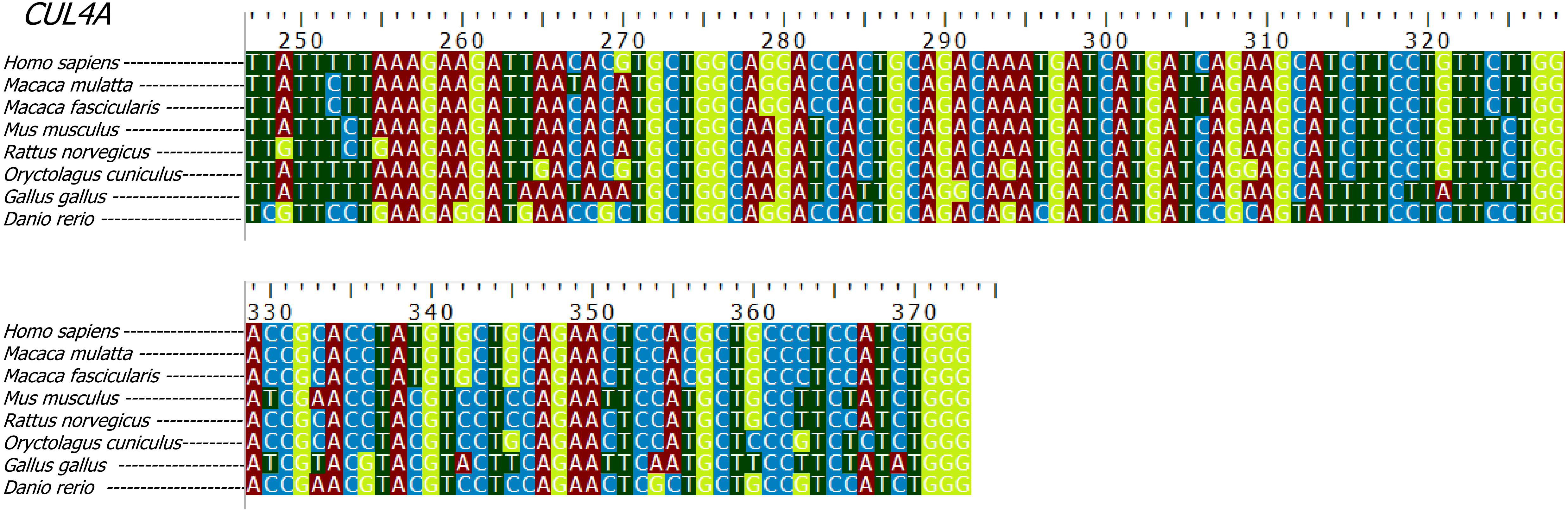
250
260
270
280
290
300
310
320
330
340
350
360
370
Homo sapiens
Macaca mulatta
Macaca fascicularis
Mus musculus
Rattus norvegicus
Oryctolagus cuniculus
Gallus gallus
Danio rerio

CUL4A

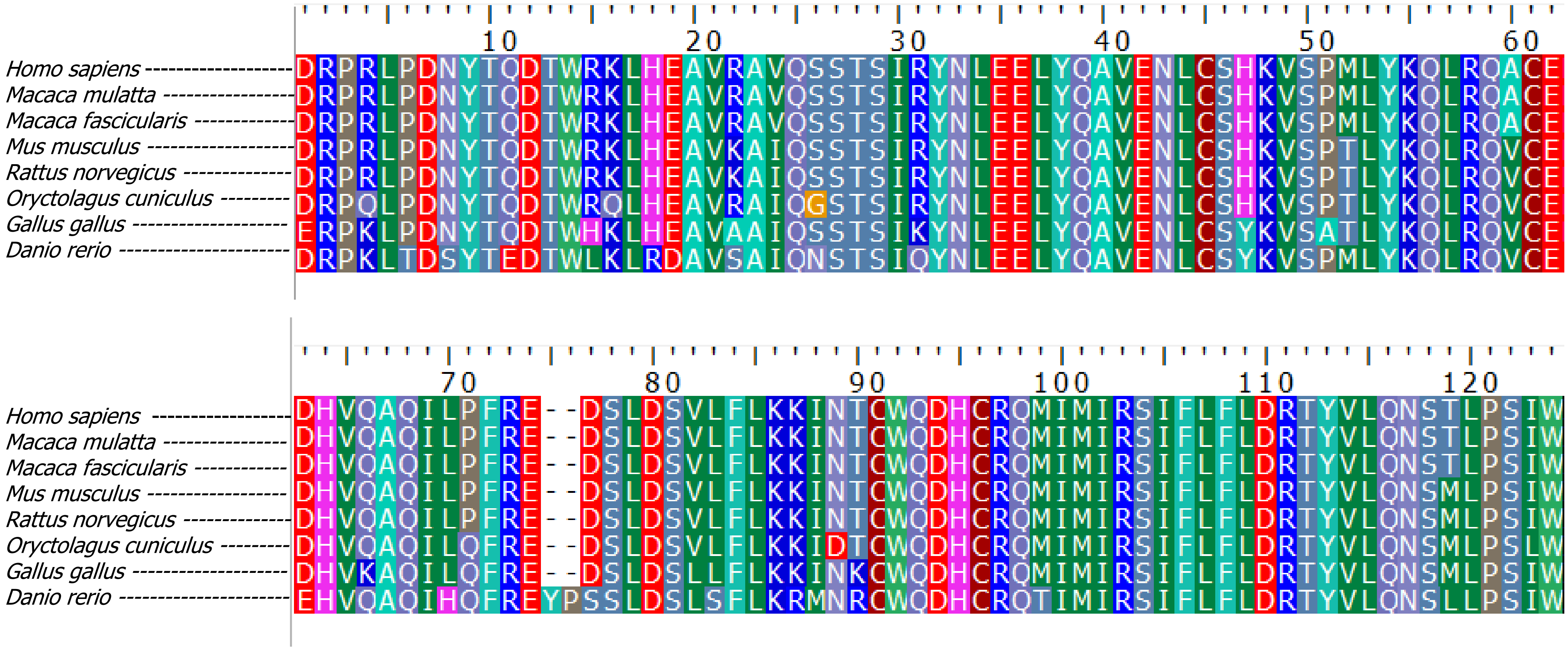

CUL4A gene – Exons 2, 3, 4, and 5 (DNA sequence) and their respective amino acids.

Supplemental File 2: Codon usage for CUL4A, IKZF1, IKZF3, and Whole Human Genome

CUL4A

TTT	F	0.49	23.7	-18	TCT	S	0.12	6.6	-5	TAT	Y	0.45	11.8	-9	TGT	C	0.44	5.3	-4
TTC	F	0.51	25	-19	TCC	S	0.19	10.5	-8	TAC	Y	0.55	14.5	-11	TGC	C	0.56	6.6	-5
TTA	L	0.15	17.1	-13	TCA	S	0.14	7.9	-6	TAA	*	0	0	0	TGA	*	1	1.3	-1
TTG	L(s)	0.14	15.8	-12	TCG	S	0.05	2.6	-2	TAG	*	0	0	0	TGG	W	1	7.9	-6
CTT	L	0.1	11.8	-9	CCT	P	0.22	6.6	-5	CAT	H	0.41	11.8	-9	CGT	R	0.05	2.6	-2
CTC	L	0.18	21.1	-16	CCC	P	0.3	9.2	-7	CAC	H	0.59	17.1	-13	CGC	R	0.12	6.6	-5
CTA	L	0.05	5.3	-4	CCA	P	0.26	7.9	-6	CAA	Q	0.17	9.2	-7	CGA	R	0.05	2.6	-2
CTG	L(s)	0.39	44.7	-34	CCG	P	0.22	6.6	-5	CAG	Q	0.83	44.7	-34	CGG	R	0.21	11.8	-9
ATT	I	0.29	15.8	-12	ACT	T	0.24	10.5	-8	AAT	N	0.44	14.5	-11	AGT	S	0.21	11.8	-9
ATC	I	0.54	28.9	-22	ACC	T	0.26	11.8	-9	AAC	N	0.56	18.4	-14	AGC	S	0.3	17.1	-13
ATA	I	0.17	9.2	-7	ACA	T	0.24	10.5	-8	AAA	K	0.41	36.8	-28	AGA	R	0.47	26.3	-20
ATG	M(s)	1	34.2	-26	ACG	T	0.26	11.8	-9	AAG	K	0.59	54	-41	AGG	R	0.12	6.6	-5
GTT	V	0.2	11.8	-9	GCT	A	0.17	9.2	-7	GAT	D	0.34	21.1	-16	GGT	G	0.11	5.3	-4
GTC	V	0.2	11.8	-9	GCC	A	0.36	19.7	-15	GAC	D	0.66	40.8	-31	GGC	G	0.42	21.1	-16
GTA	V	0.11	6.6	-5	GCA	A	0.26	14.5	-11	GAA	E	0.46	36.8	-28	GGA	G	0.32	15.8	-12
GTG	V	0.48	27.6	-21	GCG	A	0.21	11.8	-9	GAG	E	0.54	43.4	-33	GGG	G	0.16	7.9	-6

IKZF1

TTT	F	0.22	3.9 (	2)	TCT	S	0.05	5.8 (	3)	TAT	Y	0.13	3.9 (	2)	TGT	C	0.3	11.5 (	6)
TTC	F	0.78	13.5 (	7)	TCC	S	0.26	28.9 (	15)	TAC	Y	0.87	25 (	13)	TGC	C	0.7	26.9 (	14)
TTA	L	0.05	3.9 (	2)	TCA	S	0.05	5.8 (	3)	TAA	*	1	1.9 (	1)	TGA	*	0	0 (	0)
TTG	L(s)	0.07	5.8 (	3)	TCG	S	0.17	19.2 (	10)	TAG	*	0	0 (	0)	TGG	W	0	0 (	0)
CTT	L	0.1	7.7 (	4)	CCT	P	0.19	9.6 (	5)	CAT	H	0.08	3.9 (	2)	CGT	R	0.1	5.8 (	3)
CTC	L	0.29	23.1 (	12)	CCC	P	0.35	17.3 (	9)	CAC	H	0.92	46.1 (	24)	CGC	R	0.35	21.1 (	11)
CTA	L	0.05	3.9 (	2)	CCA	P	0.08	3.9 (	2)	CAA	Q	0.3	11.5 (	6)	CGA	R	0.16	9.6 (	5)
CTG	L(s)	0.45	36.5 (	19)	CCG	P	0.38	19.2 (	10)	CAG	Q	0.7	26.9 (	14)	CGG	R	0.16	9.6 (	5)
ATT	I	0.19	5.8 (	3)	ACT	T	0.26	9.6 (	5)	AAT	N	0.28	13.5 (	7)	AGT	S	0.09	9.6 (	5)
ATC	I	0.69	21.1 (	11)	ACC	T	0.42	15.4 (	8)	AAC	N	0.72	34.6 (	18)	AGC	S	0.38	42.3 (	22)
ATA	I	0.13	3.9 (	2)	ACA	T	0.05	1.9 (	1)	AAA	K	0.41	23.1 (	12)	AGA	R	0.13	7.7 (	4)
ATG	M(s)	1	36.5 (	19)	ACG	T	0.26	9.6 (	5)	AAG	K	0.59	32.7 (	17)	AGG	R	0.1	5.8 (	3)
GTT	V	0.2	9.6 (	5)	GCT	A	0.07	3.9 (	2)	GAT	D	0.39	21.1 (	11)	GGT	G	0.1	7.7 (	4)
GTC	V	0.28	13.5 (	7)	GCC	A	0.67	34.6 (	18)	GAC	D	0.61	32.7 (	17)	GGC	G	0.38	28.9 (	15)
GTA	V	0.08	3.9 (	2)	GCA	A	0.07	3.9 (	2)	GAA	E	0.32	26.9 (	14)	GGA	G	0.23	17.3 (	9)
GTG	V	0.44	21.1 (	11)	GCG	A	0.19	9.6 (	5)	GAG	E	0.68	57.7 (	30)	GGG	G	0.3	23.1 (	12)

IKZF3

TTT	F	0.36	9.8 (	5)	TCT	S	0.18	17.6 (	9)	TAT	Y	0.65	25.5 (	13)	TGT	C	0.5	17.6 (	9)
TTC	F	0.64	17.6 (	9)	TCC	S	0.08	7.8 (	4)	TAC	Y	0.35	13.7 (	7)	TGC	C	0.5	17.6 (	9)
TTA	L	0.14	9.8 (	5)	TCA	S	0.14	13.7 (	7)	TAA	*	0	0 (	0)	TGA	*	1	2 (	1)
TTG	L(s)	0.09	5.9 (	3)	TCG	S	0.04	3.9 (	2)	TAG	*	0	0 (	0)	TGG	W	0	0 (	0)
CTT	L	0.17	11.8 (	6)	CCT	P	0.29	15.7 (	8)	CAT	H	0.35	15.7 (	8)	CGT	R	0.06	3.9 (	2)
CTC	L	0.31	21.6 (	11)	CCC	P	0.36	19.6 (	10)	CAC	H	0.65	29.4 (	15)	CGC	R	0.29	19.6 (	10)
CTA	L	0	0 (	0)	CCA	P	0.29	15.7 (	8)	CAA	Q	0.29	9.8 (	5)	CGA	R	0.09	5.9 (	3)
CTG	L(s)	0.29	19.6 (	10)	CCG	P	0.07	3.9 (	2)	CAG	Q	0.71	23.5 (	12)	CGG	R	0.11	7.8 (	4)
ATT	I	0.21	7.8 (	4)	ACT	T	0.32	11.8 (	6)	AAT	N	0.59	31.4 (	16)	AGT	S	0.26	25.5 (	13)
ATC	I	0.53	19.6 (	10)	ACC	T	0.26	9.8 (	5)	AAC	N	0.41	21.6 (	11)	AGC	S	0.3	29.4 (	15)
ATA	I	0.26	9.8 (	5)	ACA	T	0.26	9.8 (	5)	AAA	K	0.53	31.4 (	16)	AGA	R	0.37	25.5 (	13)
ATG	M(s)	1	39.2 (	20)	ACG	T	0.16	5.9 (	3)	AAG	K	0.47	27.4 (	14)	AGG	R	0.09	5.9 (	3)
GTT	V	0.27	13.7 (	7)	GCT	A	0.11	5.9 (	3)	GAT	D	0.46	23.5 (	12)	GGT	G	0.16	7.8 (	4)
GTC	V	0.27	13.7 (	7)	GCC	A	0.37	19.6 (	10)	GAC	D	0.54	27.4 (	14)	GGC	G	0.24	11.8 (	6)
GTA	V	0.04	2 (	1)	GCA	A	0.37	19.6 (	10)	GAA	E	0.52	51 (	26)	GGA	G	0.36	17.6 (	9)
GTG	V	0.42	21.6 (	11)	GCG	A	0.15	7.8 (	4)	GAG	E	0.48	47.1 (	24)	GGG	G	0.24	11.8 (	6)

Whole Human Genome

UUU	F	0.46	17.6	-714298	UCU	S	0.19	15.2	-618711	UAU	Y	0.44	12.2	-495699	UGU	C	0.46	10.6	-430311
UUC	F	0.54	20.3	-824692	UCC	S	0.22	17.7	-718892	UAC	Y	0.56	15.3	-622407	UGC	C	0.54	12.6	-513028
UUA	L	0.08	7.7	-311881	UCA	S	0.15	12.2	-496448	UAA	*	0.3	1	-40285	UGA	*	0.47	1.6	-63237
UUG	L	0.13	12.9	-525688	UCG	S	0.05	4.4	-179419	UAG	*	0.24	0.8	-32109	UGG	W	1	13.2	-535595
CUU	L	0.13	13.2	-536515	CCU	P	0.29	17.5	-713233	CAU	H	0.42	10.9	-441711	CGU	R	0.08	4.5	-184609
CUC	L	0.2	19.6	-796638	CCC	P	0.32	19.8	-804620	CAC	H	0.58	15.1	-613713	CGC	R	0.18	10.4	-423516
CUA	L	0.07	7.2	-290751	CCA	P	0.28	16.9	-688038	CAA	Q	0.27	12.3	-501911	CGA	R	0.11	6.2	-250760
CUG	L	0.4	39.6	-1611801	CCG	P	0.11	6.9	-281570	CAG	Q	0.73	34.2	-1391973	CGG	R	0.2	11.4	-464485
AUU	I	0.36	16	-650473	ACU	T	0.25	13.1	-533609	AAU	N	0.47	17	-689701	AGU	S	0.15	12.1	-493429
AUC	I	0.47	20.8	-846466	ACC	T	0.36	18.9	-768147	AAC	N	0.53	19.1	-776603	AGC	S	0.24	19.5	-791383
AUA	I	0.17	7.5	-304565	ACA	T	0.28	15.1	-614523	AAA	K	0.43	24.4	-993621	AGA	R	0.21	12.2	-494682
AUG	M	1	22	-896005	ACG	T	0.11	6.1	-246105	AAG	K	0.57	31.9	-1295568	AGG	R	0.21	12	-486463
GUU	V	0.18	11	-448607	GCU	A	0.27	18.4	-750096	GAU	D	0.46	21.8	-885429	GGU	G	0.16	10.8	-437126
GUC	V	0.24	14.5	-588138	GCC	A	0.4	27.7	-1127679	GAC	D	0.54	25.1	-1020595	GGC	G	0.34	22.2	-903565
GUA	V	0.12	7.1	-287712	GCA	A	0.23	15.8	-643471	GAA	E	0.42	29	-1177632	GGA	G	0.25	16.5	-669873
GUG	V	0.46	28.1	-1143534	GCG	A	0.11	7.4	-299495	GAG	E	0.58	39.6	-1609975	GGG	G	0.25	16.5	-669768
